# Integrated Single‐Cell and TCR Profiling Reveals Protection‐Associated CD8^+^ T Cell Subsets Linked to Viral Control in PRRSV

**DOI:** 10.1002/advs.76732

**Published:** 2026-07-29

**Authors:** Can Kong, Siang Chen, Maolin Li, Meng Wang, Hailin Zhang, Bolun Zhou, Zhenhua Xie, Chen Wang, Peng Gao, Jianjun Luo, Hanchun Yang, Runsheng Chen, Dongdong Zhang, Jie Li, Jun Han

**Affiliations:** ^1^ State Key Laboratory of Epigenetic Regulation and Intervention Institute of Biophysics Chinese Academy of Sciences Beijing China; ^2^ State Key Laboratory of Veterinary Public Health and Safety Beijing China; ^3^ Key Laboratory of Animal Epidemiology of Ministry of Agriculture and Rural Affairs College of Veterinary Medicine China Agricultural University Beijing China; ^4^ Jinyu Biotechnology Co. Ltd. Hohhot China

**Keywords:** CD8^+^ T cell response, modified live vaccine (MLV), porcine reproductive and respiratory syndrome virus (PRRSV), protective immunity, single‐cell RNA sequencing (scRNA‐seq)

## Abstract

Porcine reproductive and respiratory syndrome virus (PRRSV) remains a major threat to global swine industry, yet the immune mechanisms underlying protective vaccination are incompletely understood. Here, we applied integrated single‐cell RNA sequencing and T cell receptor (TCR) profiling to characterize immune responses in a PRRSV vaccination–challenge model spanning complete, partial, and non‐protection outcomes. We identified distinct CD8^+^ T cell subsets that were selectively enriched in protected animals vaccinated with modified live vaccine (MLV) and marked by clonal expansion, strong cytotoxic transcriptional programs, and enhanced functional activity, which correlated with rapid control of viremia after challenge. In contrast, non‐protected animals accumulated dysfunctional CD8^+^ T cells expressing exhaustion‐associated markers such as CTLA4 despite partial cytotoxic signatures. Mechanistically, the protection‐associated responses were primarily driven by viral structural proteins (SP). Replacing the SP‐coding region of a heterologous strain reshaped the CD8^+^ T cell landscape from a mixed cytotoxic/exhausted profile toward a protective program, accompanied by improved clinical outcomes. Further, optimal CD8^+^ T cell activation required macrophages/monocytes‐derived innate signaling, including TLR4 and TLR8 pathways, and was enhanced by CD4^+^ T cell help. Together, our findings define protection‐associated CD8^+^ T cell states linked to viral control and provide insights for rational PRRSV vaccine design.

## Graphical abstract

PRRSV vaccine‐mediated protection is associated with clonally expanded cytotoxic CD8^+^ T cell subsets driven by viral structural proteins and supported by innate TLR4/TLR8 signaling and CD4^+^ T cell help, whereas non‐protective responses are characterized by dysfunctional exhausted‐like CD8^+^ T cells linked to poor viral control.

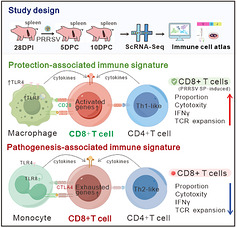

## 1 Introduction

Porcine reproductive and respiratory syndrome virus (PRRSV), an enveloped positive‐stranded RNA virus of the family *Arteriviridae* within the order Nidovirales [1], has remained a major threat to the global pork production ever since its first emergence around 35 years ago [2, 3]. Clinically, PRRSV infection primarily causes reproductive failure (e.g., stillborn, abortion, etc.) and respiratory distress, often accompanied by secondary bacterial or viral infections, leading to an annual economic losses exceeding billions of dollars in major pig production regions across North America, Asia, and Europe [4, 5, 6]. Vaccination has remained a critical measure for PRRS control in the past three decades [7, 8], and modified live vaccines (MLV), in particular, are widely used and can reduce viral tissue load and mitigate clinical symptoms [9, 10, 11, 12]. Unfortunately, PRRSV possesses an extraordinary genetic plasticity in the field [13, 14], driving continuous emergence of variants, such as the Chinese highly pathogenic PRRSV (HP‐PRRSV) [15, 16, 17, 18] and increasingly prevalent NADC30/34 strains [19, 20, 21]. Consequently, cross‐protection conferred by existing MLV against heterologous strains is often incomplete and unpredictable [22, 23, 24], striking an urgent need to define the immune determinants of vaccine‐mediated protection and to understand how viral variations impact these responses.

Despite extensive investigations, the immune determinants of protection against PRRSV have remained incompletely defined [25]. Neutralizing antibodies (NA) are typically induced at low levels and appear only after the viremia has declined [26]. Moreover, they display little activity on primary porcine alveolar macrophages (PAMs) and under certain conditions, may even contribute to antibody‐dependent enhancement (ADE) [22, 27, 28]. These observations have directed the attentions to cellular immunity as a major contributor to viral control. In retrospect, multiple PRRSV‐responsive T‐cell populations have been described, including γδ T cells, CD4^+^ Tfh, CD107A^+^ IFN‐γ^+^ T cells (CD4^+^ SP or CD4^+^ CD8^+^ DP), CD107A^+^ TNF‐α^+^ T cells (CD4^−^ CD8^−^ DN), or CD8^+^ T cells, which can produce IFN‐γ and exhibit cytotoxicity ex vivo [29, 30, 31, 32]. However, much of the existing evidence are mainly derived from analyses of peripheral blood mononuclear cells (PBMCs) [29, 33, 34], which do not capture the tissue context of viral replication or antigen presentation. In addition, the flow cytometry‐based (FACS) analysis in pigs remain constrained by limited antibody availability and incomplete definition of porcine T cell population. Consequently, the contribution of individual T cell subsets to PRRS protective immunity has remained controversial or unresolved.

Recent advances in single‐cell transcriptomics (scRNA‐seq) and T‐cell receptor profiling offer an opportunity to address these limitations by enabling simultaneous characterization of immune cell composition, activation status, and clonal dynamics in relevant organs at high resolution [35, 36]. Here, by combining an immunization‐challenge pig model spanning clinically complete, partial, and absent protection with single cell sequencing, clonal tracking of T cell receptor repertoires, and functional assays of immune cells, we investigated the quantity, quality, and heterogeneity of T cell responses to PRRSV in the spleen. We choose spleen for analysis because it is a major secondary lymphoid organ against blood‐borne pathogens and provides an informative window into systemic antiviral immunity [37], in particular given that PRRSV infection is characterized by prolonged viremia [38]. Thus, the spleen provides a biologically relevant setting to capture systemic immune dynamics associated with viral control.

Our analyses identify distinct CD8^+^ T cell subsets whose expansion and functional state are strongly associated with clinical protection outcomes. We further show that viral genetic variation is linked to reshaping of the CD8^+^ T cell landscape, and that protection‐associated states are enriched following structural protein (SP) exchange. In addition, we define an exhaustion‐associated signature linked to failure in non‐protective settings, map the cellular interactions that drive effective priming, and uncover an unexpected requirement for macrophages/monocytes help via TLR4 and TLR8 signaling, together with CD4^+^ T cell support to achieve effective CD8^+^ T cell activation. Together, these findings provide a systems‐level view of immune cell states associated with viral control and offer insights into cellular correlates of protection in PRRSV infection.

## 2 Results

### 2.1 Establishment of a Pig Model for Homologous and Heterologous Protection

**FIGURE 1:**
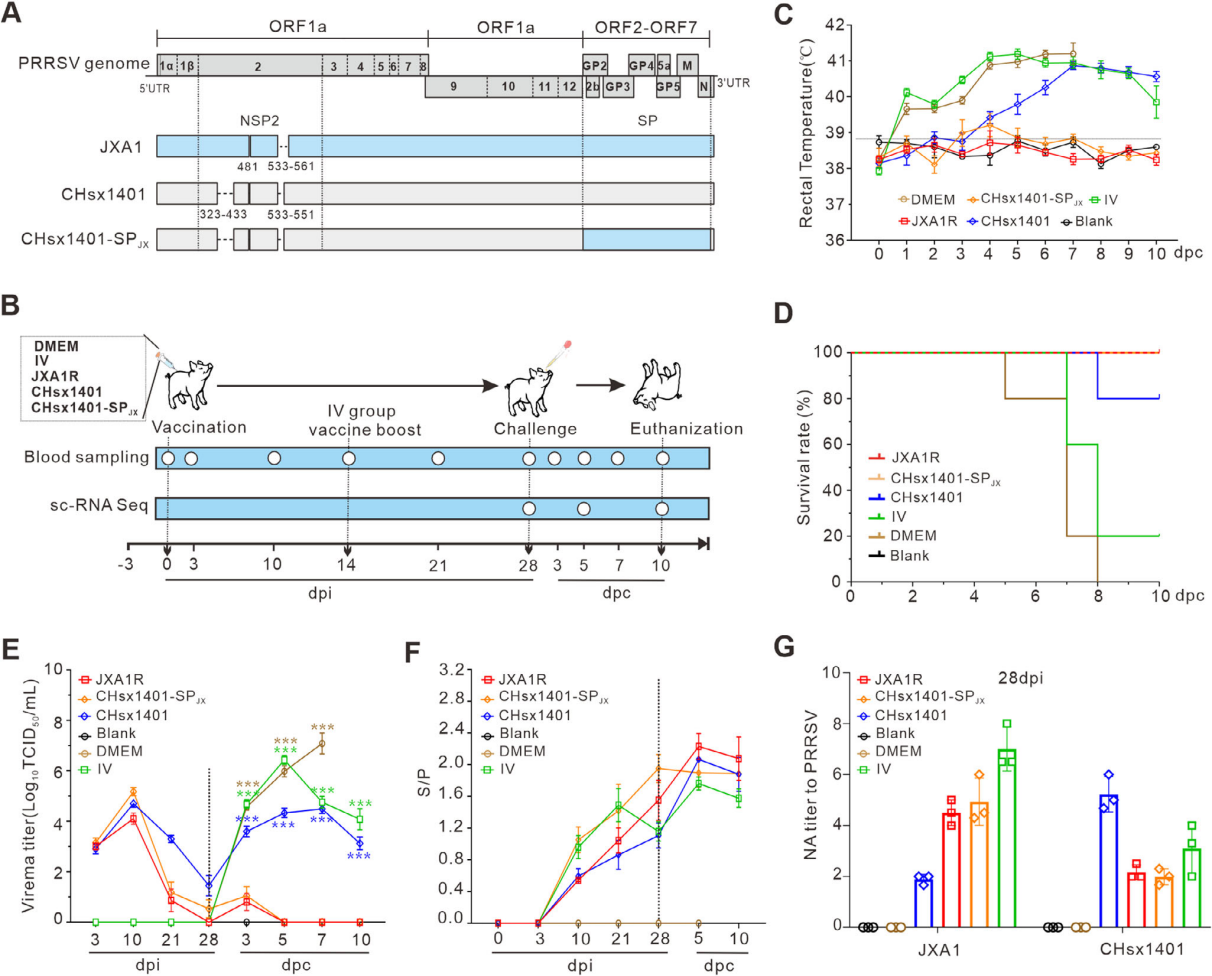
A SPF pig model for homologous and heterologous immunity. (A) Genome organization and difference of PRRSV strains. (B) Experimental timeline for immunization‐challenge animal experiment. (C) Pig rectal temperature dynamics following challenge. (D) Animal survival curve following challenge. (E) PRRSV viremia kinetics before and after challenge. (F) Total serum antibody responses by an IDEXX HerdChek PRRS X2 ELISA kit. A sample/positive value (S/P) of ≥0.4 was regarded as seroconversion. (G) The serum neutralization titer (NA) against the JXA1 and CHsx1401 at 28 dpi. Statistical analysis was performed by two‐tailed Student's t‐test, and the error bars indicate means ± standard error of mean (SEM). Asterisks (*) indicate the statistical significance: *, *p* < 0.05; **, *p* < 0.01; ***, *p* < 0.001.

To dissect immune determinants associated with homologous and heterologous protection, we established an immunization‐challenge model by using three immunogens: a commercially available MLV vaccine JXA1R, a derivative of the HP‐PRRSV strain JXA1 (PRRSV‐2 lineage 8); a low‐virulent NADC30‐like strain CHsx1401 (PRRSV‐2 lineage 1); and a chimeric virus CHsx1401‐SP_JX_, in which the structural protein (SP)‐coding region was replaced with that from HP‐PRRSV JXwn06 (PRRSV‐2 lineage 8) (Figure 1A). Controls included the inactivated form (IV) of JXA1R, DMEM, and an untreated blank group. One‐month old SPF pigs were randomly assigned to 6 groups (>16 pigs/group, except the blank group with *n* = 3). Animals were immunized intramuscularly at a dose of 2 × 10^5^ TCID_50_ of the respective immunogens or DMEM; the blank group left untreated, whereas the IV group received two immunizations at a 14‐day interval. At 28 days post immunization (dpi), all the pigs except the blank were challenged with HP‐PRRSV strain JXA1. For scRNA‐seq analysis, three pigs per each group were euthanized at 28 dpi, 5 dpc, and 10 dpc (Figure 1B).

Following the challenge, distinct clinical outcomes were observed across groups. Animals immunized with JXA1R or CHsx1401‐SP_JX_ showed no overt clinical symptoms (Figure 1C,D; Figure S1A). In contrast, the IV group developed the earliest onset of fever (>40°C) and progressive clinical signs, including coughing, sneezing, cyanosis, depression, and loss of appetite (Figure 1C; Figure S1A), with 80% mortality by 10 dpc, the trial termination date, with the remaining animals severely debilitated (Figure 1D). The CHsx1401 group showed delayed disease onset, (hyperthermia from 6 dpc), intermediate clinical severity, with 20% mortality rate and persistent fever in survivors, indicating incomplete protection (Figure 1C,D; Figure S1A). All animals in DMEM group died before 8 dpc, whereas the blank group remained healthy throughout (Figure 1D).

These clinical observations were also consistent with virological and pathological measurements. JXA1R and CHsx1401‐SP_JX_ groups exhibited lower viremia that declined rapidly after challenge (Figure 1E), together with markedly reduced viral loads across tissues, whereas IV and DMEM groups showed high viral burden (Figure S1F,G). Lung pathology scores followed a similar trend (Figure S1B–E). Notably, although PRRSV‐specific antibodies became detectable from 10 dpi (Figure 1F), neutralizing antibody titer remained low (<1:8) at 28 dpi (Figure 1G), arguing against it as the primary protection determinant in this model.

Based on these outcomes, we thus successfully established a vaccination‐challenge pig model that includes clinically complete protection (JXA1R and CHsx1401‐SP_JX_), partial protection (CHsx1401), and non‐protection (IV and DMEM) to allow further dissection of cellular immune features associated with differential protection against PRRSV.

### 2.2 Single‐Cell Immune Cell Landscape of Spleen

**FIGURE 2:**
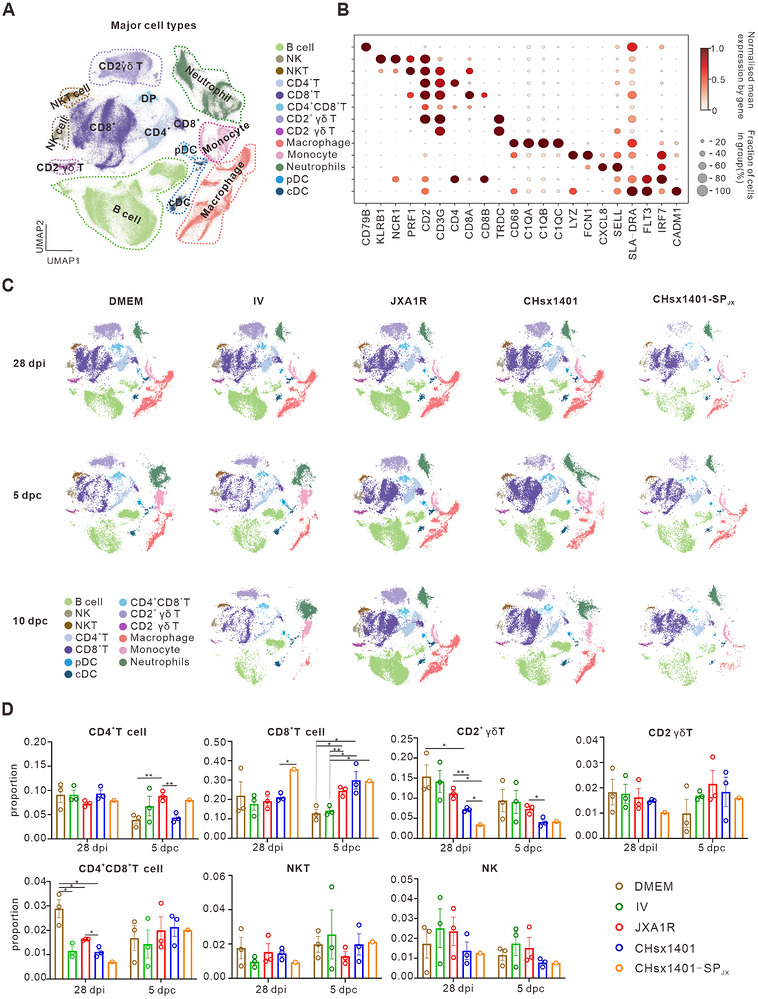
Porcine spleen immune cell atlas and compositional changes following PRRSV challenge. (A) Uniform manifold approximation and projection (UMAP) of immune cells from all experimental groups. A total of 289 186 splenic cells pooled from 41 pigs were analyzed, and the different colors indicate the major cell types. (B) Marker‐based definition of the different cell types. Specific markers used to delineate the individual cell type are indicated, and their expression levels represent group‐wise mean log‐normalized values scaled from 0 to 1 for each gene. (C) The proportion of individual spleen cell types at 28 dpi and 5 dpc. (D) UMAP of immune cells at indicated days of experiment. Statistical analysis was performed by two‐tailed Student's t‐test, and the error bars indicate means ± standard error of mean (SEM). Asterisks (*) indicate the statistical significance: *, *p* < 0.05; **, *p* < 0.01; ***, *p* < 0.001.

We next employed 3′ scRNA‐seq to characterize immune cell dynamics at indicated 28 dpi, 5 dpc, and 10 dpc). Three pigs per group were randomly sampled at each time point. The only exceptions were the IV group at 10 dpc, in which only two piglets remained available due to mortality, and the DMEM group at 10 dpc, from which no samples were collected because all pigs had succumbed prior to this time point. For single‐cell sequencing, spleen cells were sequenced individually for each animal, while pooling was performed only for the CHsx1401‐SP_JX_ group, which was included primarily as a confirmatory control. After quality control, a total of 289,186 splenic cells from 41 pigs were retained, with a median of 6760 transcripts and 2379 detected genes per cell. To comprehend the overall cell atlas in the spleen, we combined all the sequencing information into one using the method of graph‐based clustering of uniform manifold approximation and projection (UMAP). With *CD45* as a marker (all immune cells express this molecule), the immune cells could be classified into 24 clusters that were further grouped into 12 cell types by manual annotation based on the expression of known markers (Figure 2A,B).

Across time and groups, differential abundance analysis revealed that CD8^+^ T cells were the only cell lineage showing a clear proportional increase in completely/partially protected groups following PRRSV challenge (notably at 5 dpc), whereas this expansion was less evident in non‐protected DMEM group (Figure 2C,D). Further flow cytometry analysis at the population level showed the similar trend for CD8^+^ T cells in protected animals following challenge (Figures S2 and S3). This selective expansion suggests that CD8^+^ T cell responses may be a major correlate of protection.

### 2.3 Protection Is Associated With Specific CD8^+^ T Cell Subsets With Non‐Exhausted Effector Programs

**FIGURE 3:**
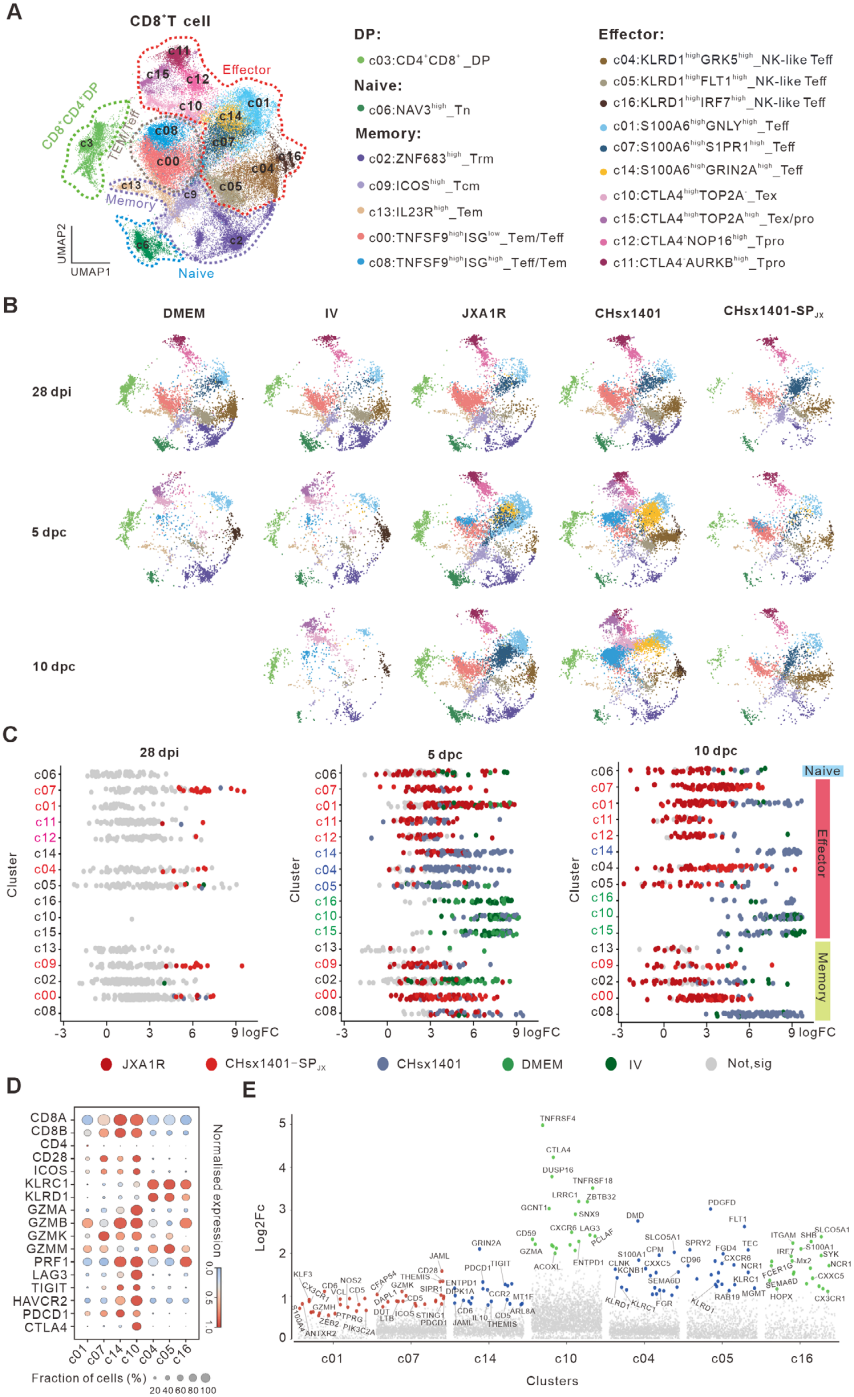
CD8^+^ effector T cells are significantly enriched in immune protected groups following challenge. (A) UMAP plot of CD8^+^ T cell clusters (*n* = 60,101). (B) Temporal dynamic changes of T cell subpopulations by UMAP plot. (C) Abundance analysis of significantly enriched CD8^+^ clusters in different groups at indicated time points. Each dot represents a MiloR neighborhood. The logFC (fold of changes, FC) reflect the abundance change of subclusters within each T cell clusters in each experimental group that was normalized against that of DMEM at 28 dpi. Colored neighborhoods indicate significant differential abundance (FDR < 0.1). (D) Dot plot showing normalized mean expression levels of characteristic genes for effector CD8^+^ T clusters. The expression levels represent group‐wise mean log‐normalized values scaled from 0 to 1 for each gene. Dot color and size indicate normalized mean expression and the percentage of expressing cells, respectively. (E) Volcano map of differentially expressed genes of individual cell populations associated with complete immune protection (c07, c01, c11, and c12), partial protection (c14, c04, and c05), and non‐protection (c10, c15, and c16).

To resolve CD8^+^ T cell heterogeneity, we reclustered of 59,136 CD8^+^ T cells using unsupervised cell clustering and identified 17 transcriptionally distinct subclusters (Figure 3A). These cluster were annotated based on differentially expressed genes associated with cytotoxic activity, proliferation state, and lineage‐defining transcriptional programs (Figure S4A). Several patterns emerged with clear group specificity. Specifically, CD8^+^ memory cell clusters (e.g., c09 and c00) were exclusively enriched in the protected group. Effector CD8^+^ T cells could be divided into two populations based on *CD28* mRNA expression, namely *CD28* ^high^ T cells with expression, and *CD28* ^low^ NK‐like T cells (Figure 3A–D). Following viral challenge, NK‐like T cell clusters (c04 and c05) were notably enriched in the completely/partially protected groups without overt clinical signs at 5 dpi, whereas c16, marked by stronger innate immune activation, was restricted to clinically diseased animals. Among *CD28* ^high^ effector CD8^+^ T cells, clusters c01, c07, c11, and c12 expanded prominently in the JXA1R group after challenge, but much less represented in non‐protected groups (IV and DMEM), which instead showed enrichment of clusters (c10 and c15) expressing inhibitory or exhaustion‐associated markers such as *CTLA4* in the time‐course analysis (28 dpi, 5 dpc, and 10 dpc) (Figure 3B–D). The partially protected CHsx1401 group displayed an intermediate and unstable phenotype, retaining substantial c01/c11/c12 populations but with significantly reduced representation of c07, accompanied by compensatory increase of effector c14 cluster and a concurrent rise of exhaustion‐associated clusters c10/c15 (Figure 3B,C). Notably, exchanging the SP region of CHsx1401 with that of HP‐PRRSV (CHsx1401‐SP_JX_) resulted in immune responses shifted toward a CD8^+^ T cell landscape more similar to that observed in the JXA1R group (Figure 3B), consistent with the restored clinical protection, implicating c01/c07/c11/c12 as protection‐linked subsets. This observation also links SP‐dependent viral genetic variation with alterations in CD8^+^ T cell states.

Gene expression programs further distinguished these CD8^+^ T cell states. Differential expression and pathway analyses showed that protection‐associated subsets (e.g., c01 and c07) were enriched for antigen presentation and T cell activation pathways and expressed high levels of cytotoxic effectors including *GZMB*, *GZMK*, and *GZMM* (Figure 3D,E; Figure S4C). In contrast, clusters enriched in non‐protected groups (e.g., c10) showed increased expression of inhibitory/exhaustion‐associated genes (e.g., *CTLA4*, *LAG3*, *IL‐10*), together with signatures consistent with cellular stress and dysfunction, including pathways related to autophagy, necroptosis and apoptosis, despite retaining expression of cytotoxic transcripts such as *GZMA*/*GZMB* (Figure 3D,E; Figure S4C). The CHsx1401 group again exhibited an intermediate transcriptional profile, reflecting a transitional immune state (Figure 3D,E; Figure S4C), consistent with its partial protection phenotype. Collectively, these results suggest that protection is associated not simply with the presence of cytotoxic gene expression, but with distinct CD8^+^ T cell states characterized by coordinated effector programs and reduced inhibitory/exhaustion features.

### 2.4 CD4^+^ T Cell Polarization Differs Across Protection States, but CD8^+^ T Cells Exhibit Dominant Cytotoxic Features

**FIGURE 4:**
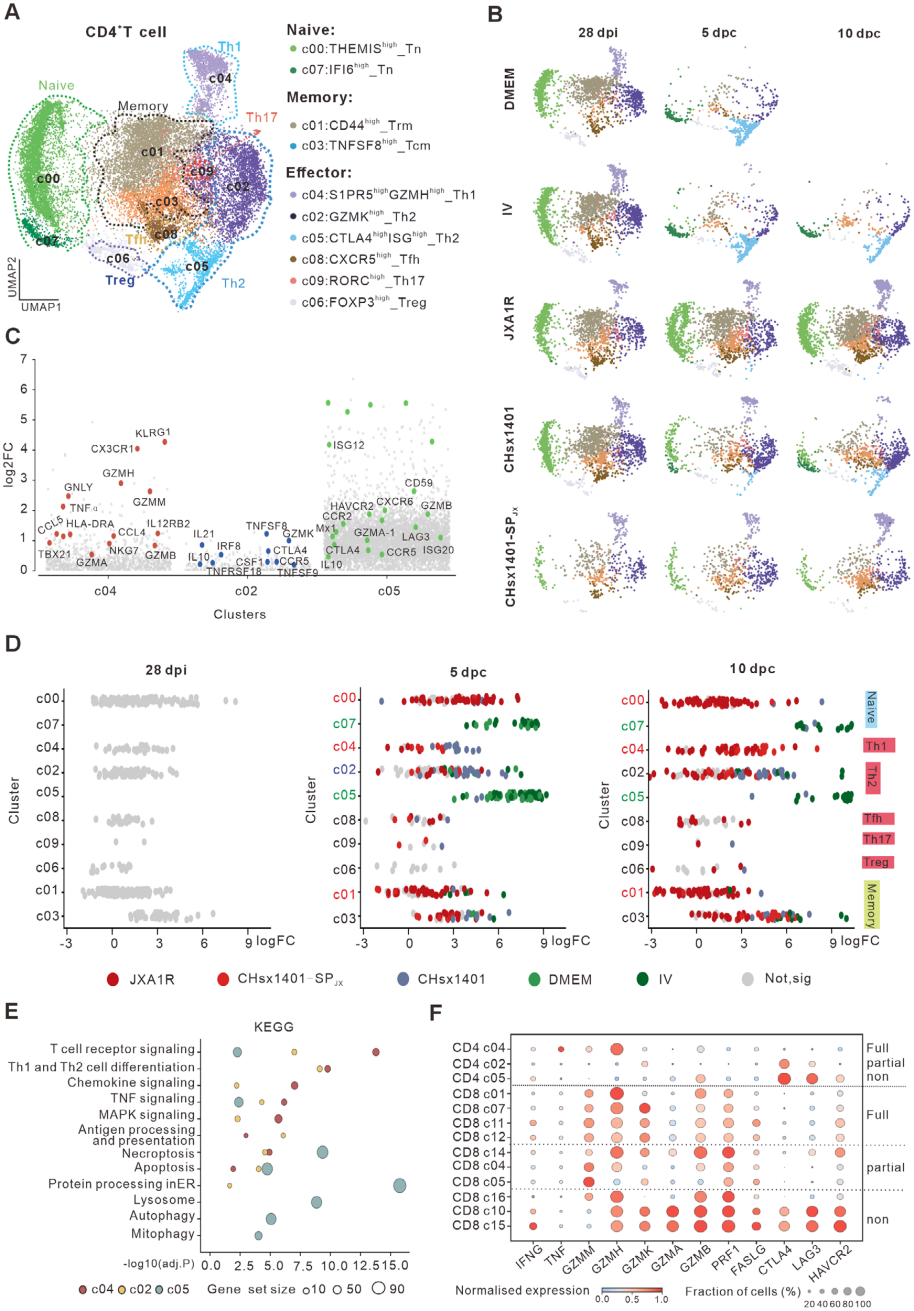
Distinct CD4^+^ T cell subsets are differentially enriched in protected versus non‐protected pigs. (A) UMAP plot of CD4^+^ T clusters (*n* = 21,814). (B) Temporal dynamic changes of CD4^+^ T cell subpopulations. (C) Volcano map of differentially expressed genes of CD4^+^ effector subsets (c02, c04, and c05). (D) Abundance analysis of significantly enriched CD4^+^ clusters in different groups at indicated time points. (E) The KEGG enrichment analysis of differentially upregulated genes in CD4^+^ effector subsets (c02, c04, and c05). (F) Scaled expression levels by dot plot of cytotoxicity‐related genes in CD4^+^ and CD8^+^ effector subclusters. Expression values represent group‐wise mean log‐normalized expression scaled from 0 to 1 for each gene. Dot color and size indicate normalized mean expression and the percentage of expressing cells, respectively.

Reclustering of 20,144 CD4^+^ T cells led to identification of 10 transcriptionally distinct subclusters (Figure 4A; Figure S4B). Temporal dynamics via UMAP (Figure 4B) and quantitative analysis via MiloR (Figure 4D) revealed divergent trajectories across experimental groups. In the non‐protected groups (IV and DMEM), there was a prominent loss of naïve (c00), memory (c01), and effectors (c04/c08), accompanied by a clear emergence/increase of alternative naïve/effector/memory states (e.g., c07 naïve; c05 effector). These changes are consistent with a shift in CD4^+^ T cell composition toward immune programs less supportive of effective cellular immunity (Figure 4B,D). In contrast, the protected groups (JXA1R and CHsx1401‐SP_JX_) showed an increase of c00 (naïve), c01 (memory), and notably c04 (effector) populations (Figure 4B,D). Particularly, cluster c04 exhibited features consistent with a Th1‐like program, including upregulation of genes associated with immune activation (Figure 4C), consistent with the helper function.

The CHsx1401 group displayed an intermediate profile. Although c04 increased at 5 dpc, it declined by 10 dpc, coinciding with disease progression (Figure 4B,D). KEGG pathway enrichment further distinguished effector subsets: c04 in protected groups was enriched for pathways related to immune activation, whereas c05 and c02 showed enrichment patterns associated with immune suppression (Figure 4E). Finally, comparative analysis of expression of cytotoxicity‐related genes revealed that the CD8^+^ effector clusters expressed substantially higher levels of cytotoxic/activation associated genes modules than CD4^+^ subsets (Figure 4F), supporting a model in which CD4^+^ T cells primarily provide help, whereas CD8^+^ T cells are more directly associated with cytotoxic effector functions.

### 2.5 CD8^+^ T Cells in Protected Groups Exhibit Higher Cytotoxicity and Require the Help From CD4^+^ T Cells

To link transcriptional state with function responses, we performed IFN‐γ ELISpot assay on splenocytes at 28 dpi (pre‐challenge) and 5 dpc (early post‐challenge). The 28 dpi time point was to evaluate the baseline functional competence prior to viral challenge, while 5 dpc represents an early phase at which the viremia was almost cleared in protected groups but continue to rise in non‐protected groups was being on rise. The isolated spleen cells were in vitro stimulated with whole virions (JXA1, CHsx1401, CHsx1401‐SP_JX_) or PBS as an unstimulated negative control.

**FIGURE 5:**
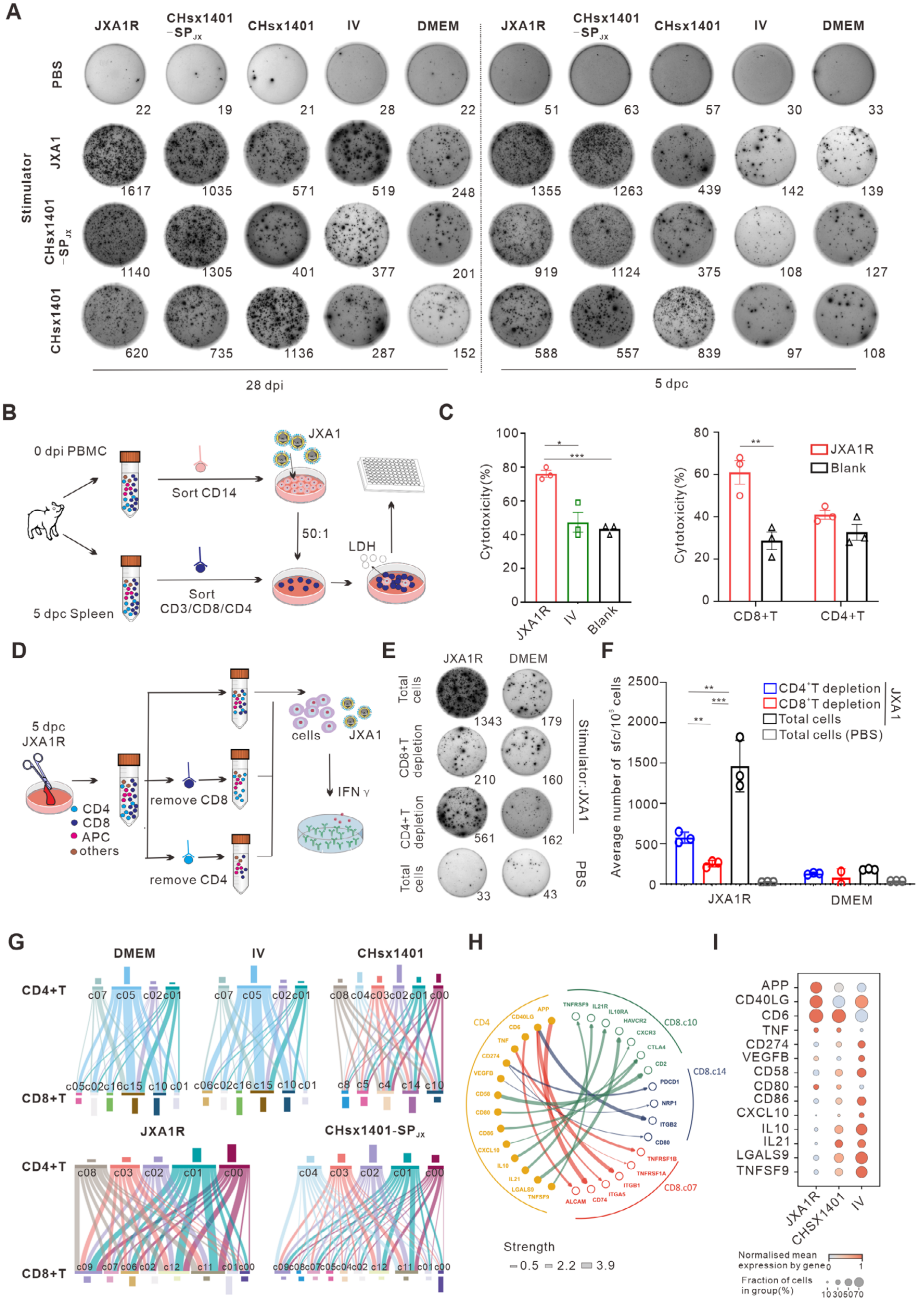
CD8^+^ T cells in protected groups exhibit robust killing ability and requires assistance from CD4^+^ T cells for maximal activity. (A) ELISpot assay of IFN‐γ secretion. (B) Protocol for measuring cellular cytotoxicity of CD4^+^ and CD8^+^ T cells. Monocytes from peripheral blood from 3 pigs at 0 dpi of each experimental group as indicated were sorted with antibodies to CD14, whereas spleen T cells were isolated with mouse antibodies to CD3 or CD8α/β or CD4 from the same pigs at 5 dpc using magnetic beads. (C) T cell cytotoxicity. (Left) Comparison of T cell cytotoxicity from JXA1R, IV, and Mock groups; (Right) the cytotoxicity of CD4^+^ and CD8α^+^ T cells from JXA1R and mock groups. (D–F) The loss‐of‐function assay to assess contribution of CD4^+^ and CD8α^+^ T cells by ELISpot assay. (D) The experimental protocol for removing CD4^+^ or CD8α^+^ T cells from spleen cell mixture; (E) Representative pictures of ELISpot. (F) Quantitative analysis. (G) Analysis of the communications between CD4^+^ and CD8^+^ T cells within each experimental group using CellPhoneDB. The proportion of each cell subpopulation among total T cells in their respective groups is represented by bar graphs. The communication strength was calculated using CD4^+^ as the ligand and CD8^+^ as the receptor, and the width of connecting lines between subpopulations represents the number of the ligand‐receptor communications. (H) The dominant communication factors in protection‐related or diseased‐related clusters. (I) Expression of communication factors in CD4^+^ T clusters. Statistical analysis was performed by two‐tailed Student's t‐test and error bars indicate means ± standard error of mean (SEM). Asterisks (*) indicate the statistical significance: *, *p* < 0.05; **, *p* < 0.01; ***, *p* < 0.001.

At 28 dpi, restimulation with JXA1 elicited strong IFN‐γ responses in the JXA1R group but much weaker responses in the CHsx1401 group. Notably, incorporation of the JXA1‐derived structural protein (SP) region into CHsx1401 (CHsx1401‐SP_JX_) enabled significantly enhanced IFN‐γ responses under JXA1 re‐stimulation conditions (Figure 5A left; Figure S5A). This overall recall response again is consistent with the role of SP region in protective immunity. Conversely, CHsx1401 restimulation elicited much stronger IFN‐γ secretion in CHsx1401‐immunized animals than that in JXA1R‐immunized animals, whereas SP replacement (CHsx1401‐SP_JX_) significantly reduced this response (Figure 5A left; Figure S5A), consistent with the notion that antigenic variation can reshape the immune landscape. For the PBS control, it consistently gave minimal background signals across all groups, arguing against contamination or technical artifacts. In addition, the magnitude of IFN‐γ responses following whole virus stimulation in the DMEM group remained substantially lower than those observed in immunized groups. These virus‐associated background responses likely reflect background activation of innate or innate‐like immune cells and antigen‐presenting cells triggered by viral components during in vitro stimulation, although technical contributions cannot be completely excluded.

At 5 dpc, similar overall patterns were observed, although group‐specific differences became more apparent. Following JXA1 restimulation, CHsx1401 animals showed reduced IFN‐γ relative to 28 dpi (Figure 5A right; Figure S5B), consistent with transcriptional features (e.g., exhaustion markers) associated with reduced effector function. Notably, although CHsx1401‐SP_JX_ conferred complete clinical protection, the IFN‐γ response to JXA1 restimulation did not reach the level observed in the JXA1R group, suggesting that additional components beyond the SP region (e.g., replicase proteins) contribute the magnitude of recall responses.

We then measured the cellular cytotoxicity using a lactate dehydrogenase (LDH) release assay. Splenic T cells from JXA1R, IV, and blank groups at 5 dpc were isolated and cocultured with PRRSV‐infected autologous CD14^+^ monocytes isolated from the same pig before immunization (Figure 5B). The T cells from the JXA1R group exhibited substantially higher cytotoxicity than those from IV or mock controls (Figure 5C left). The individual functional competency of CD4^+^ and CD8^+^ T cells in the JXA1R group was further evaluated by sorting out the respective population with antibodies to CD4 or CD8α. The coculture assay confirmed that the CD8^+^ T cells mediated a stronger killing than CD4^+^ cells (Figure 5C, right; Figure S5C,D). As CD8α is also expressed on some other cells (although in low proportion) (Figure 2B) [39, 40], including activated CD4^+^ T cells, NK cells, and γδ T cells, we used antibodies to CD8β for further analysis (Figure S6A). Overall, CD3^+^CD8β^+^ T cells exhibited cytotoxic activity similar to that observed using CD3^+^CD8α^+^ T cells in the LDH killing assay (Figure 5C, right; Figure S6B), indicating that the overall functional conclusions are not obviously dependent on the choice of CD8 marker.

We next assessed the relative contribution of CD4^+^ and CD8^+^ T cells to IFN‐γ secretion using depletion assay with respective antibodies (Figure 5D). Depletion of CD8^+^ T cells with antibodies to CD8α resulted in a substantial reduction (∼83%) in IFN‐γ secretion compared to the non‐depletion control (Figure 5E,F; Figure S5E), and further validation with antibodies to CD8β yielded similar results (∼80%) (Figure S6C,D), thus indicating a major contribution from CD8^+^ T cells. Depletion of CD4^+^ T cells also led to a marked decrease (∼53%) in IFN‐γ production, suggesting that CD4^+^ T cells contribute to optimal CD8^+^ T cell responses rather than acting as primary producers of IFN‐γ. Consistent trends were observed in cytotoxicity assays (Figure S5F).

To further explore potential interactions, cell‐cell communication analysis using CellPhoneDB revealed distinct interaction patterns between CD4^+^ and CD8^+^ T cell subsets across different groups (Figure 5G). In particular, the CD4^+^ T cell cluster c05, a subpopulation expressing high level of CTLA4, showed unique and preferential interactions with exhausted CD8^+^ T cell clusters c15 and c10 in the non‐protected groups (DMEM and IV). In contrast, multiple CD4^+^ T cell clusters (c01, c02), enriched for activating ligands such as *TNF*, *CD40LG, CD6*, *APP*, and *CD80* (Figure 5G,H), exhibited much stronger interactions with protaction‐associated CD8+ T cells subsets (c01, c07, c11 and c12) in protected groups (JAX1R and CHsx1401‐SP_JX_). In addition, the CD4^+^ T cell clusters c00 and c03, which also expressed activating signals, displayed unique communication patterns that were preferentially observed in protected groups but largely absent in non‐protected groups. Overall, the non‐protected groups exhibited increased interactions associated with inhibitory/exhaustion signals (e.g., *IL‐10*, *IL‐21*, *CXCL10*, *LGALS9*), particularly involving CD8^+^ T cell clusters with features of exhaustion (Figure 5G–I), whereas the protected groups were characterized by enhanced activating interactions. The CHsx1401 group exhibited an intermediate interaction profile (Figure 5G–I). Although the phonotypes for its CD4^+^ T cells largely resembled those observed in protected groups, they also exhibited increased crosstalk with exhausted CD8^+^ T cell cluster c10 (Figure 5G).

Together, these results indicate that CD8^+^ T cells are strongly associated with cytotoxic effector activity, while CD4^+^ T cells appear to support and enhance the potency of CD8^+^ T cells, contributing to the differences in immune outcomes across protection states.

### 2.6 Protection‐Associated CD8^+^ T Cell Subsets Represent the Major Clonally Expanded T Cell Populations

**FIGURE 6:**
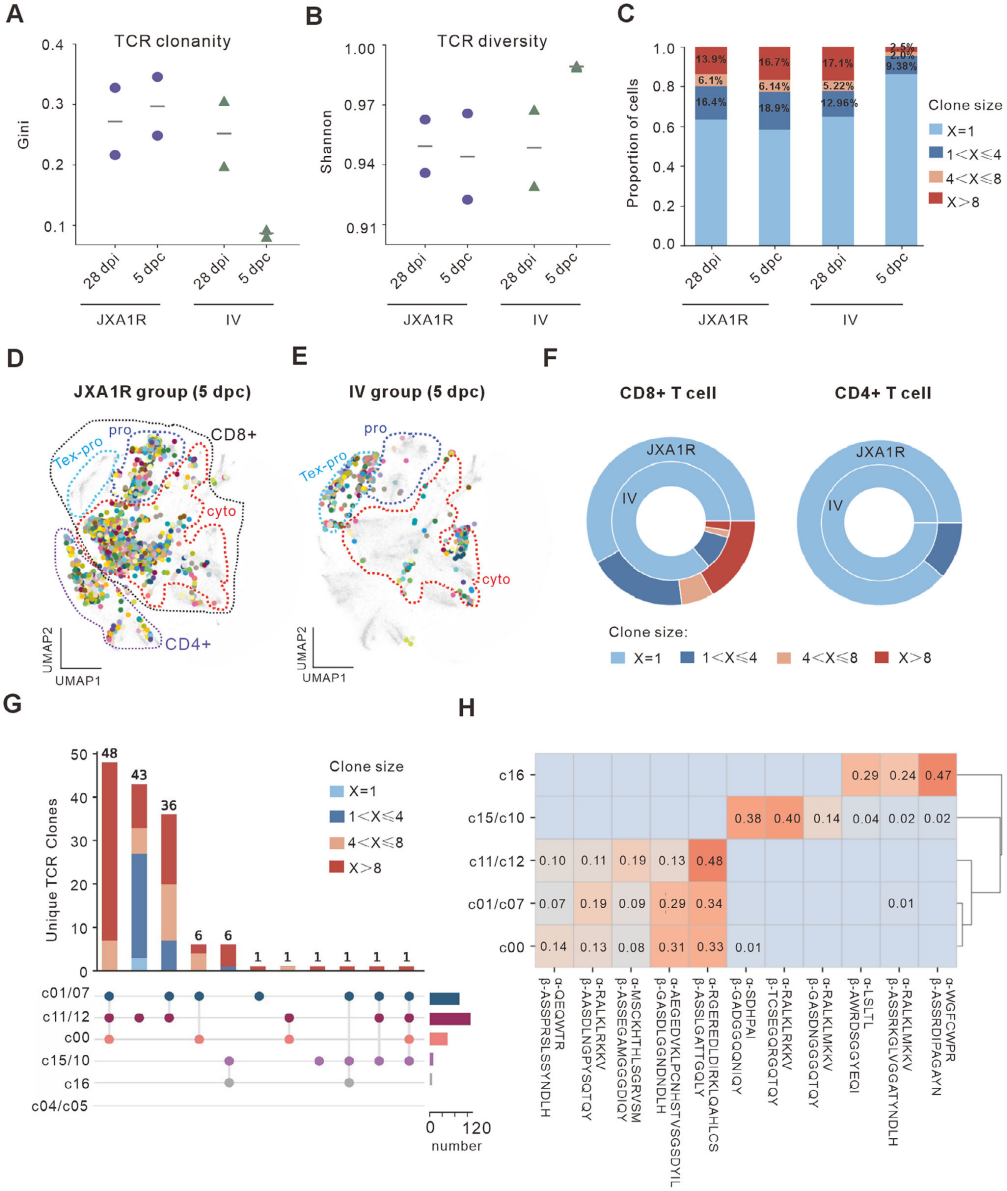
CD8^+^ T cells are the major PRRSV‐specific T cell types following challenge. (A) Analysis of TCR clonality of the JXA1R and IV groups at 28 dpi and 5dpc by Gini index. (B) Analysis of TCR diversity of the JXA1R and IV groups at 28 dpi and 5dpc by Shannon index. (C) Proportion of cell numbers from different clone sizes in JAX1R and IV groups at indicated time points. X = 1 represents non‐clonality, X = 2 represents low clonality, 2 < X ≤ 4 represents moderate clonality, and X > 8 represents high clonality. (D, E) Distribution of specific T cell clones in JXA1R and IV groups at 5 dpc. Colored points representing cells with a clone size ≥ 2, and different cell types were indicated by dashed circles. (F) Pie chart showing the clonality proportion of CD8^+^ and CD4^+^ T cells from JXA1R and IV groups at 5 dpc. (G) The UpSet plot of TCR clone size distribution in protection‐associated (c00, c01/c07, c11/c12) and disease‐associated cell clusters (c 10, c15, c16). Each dot represents the presence of the corresponding TCR clones in the indicated cell clusters. For example, in the first column, the three dots in the row mean that the 48 types of TCR clones are shared by the three cell clusters (c01/07, c11/12, and c00). (H) TCR CDR3 usage frequency. The top 6 most frequently used CDR3 sequences for each cluster are shown with a threshold value of >0.01.

To characterize the clonal architecture of T cell responses, we performed 5’ single cell TCR sequencing (5’ scTCR‐seq) on spleen samples from IV and JXA1R groups at 28 dpi and 5 dpc, generating a dataset comprising 79,080 cells (Figure S7A,B). TCR reads were assembled into contigs using the Cell Ranger V(D)J pipeline, and the CDR3 sequence was identified based on conserved alignment boundaries. Clonal expansion was quantified using Gini index (clonality) and Shannon index (diversity) (Figure 6A,B), where the same clonotype is defined as T cells sharing identical paired CDR3α and CDR3β sequences.

At 5 dpc, the JXA1R group exhibited significantly higher clonality and lower diversity than the IV group (Figure 6A,B), indicating transition from a broadly distributed TCR repertoire to a directional, focused clonal expansion. Consistently, the proportion of highly expanded clones (X>8) increased from 13.9% to 16.7% in JXA1R animals following challenge, whereas this population in the IV group underwent a significant shrink from 17.1% to 2.5% (Figure 6C), suggesting impaired expansion of antigen‐responsive T cells under non‐protective conditions.

We next analyzed the distribution of T cells with clonal expansion properties at 5 dpc (Figure 6D,E). In the JXA1R group, the high clonality was predominantly enriched within CD8^+^ cytotoxic T lymphocytes (CTLs), whereas CD4^+^ T cells largely remained in low‐clonality states (Figure 6D–F). In contrast, the IV group showed minimal clonal expansion in CD8^+^ T cells and no evident expansion for CD4^+^ T cells (Figure 6D–F), consistent with the limited ability to secret IFN‐γ, suggesting that the cellular immunity in this group is very much restricted. These results suggested a dominant role for CD8^+^ T cells in antigen‐driven expansion during protective immunity.

To further link clonal expansion with the earlier identified T cell clusters, we integrated the 3' and 5' single‐cell sequencing datasets based on differentially expressed genes and overall gene expression profiling. The high clonal T cell populations in the 5' dataset precisely matched to protection‐associated CD8^+^ T cell clustersin the 3' dataset (c00, c01, c07, c11, and c12), while the low clonality populations in the 5' dataset aligned with the diseased‐associated CD8^+^ T cell clusters (c15, c16, and c10) (Figure S7C,D). This correspondence indicates that the afore defined protection‐associated subsets represent the major clonally expanded effector populations.

Analysis of TCR usage further revealed distinct repertoire features between protective and non‐protective states. The majority of highly clonal TCRs (120/129) at dpc were shared among protection‐associated cell clusters (c01, c07, c11, c12, and c00) (Figure 6G), whereas only a limited number of shared TCRs (9 total) were observed among disease‐associated clusters (Figure 6G), indicating divergent TCR selection patterns. Moreover, comparison of CDR3 usage frequency among the top six most frequently used TCRs showed that there was no overlap between protection‐associated and disease‐associated clusters (Figure 6H), supporting the existence of distinct antigen‐driven selection regimes. For example, the dominant clonotypes in protected animals included the CDR3α sequence RGEREDLDIRKLQAHLCS paired with CDR3β sequence ASSLGATTGQLY, whereas non‐protected animals exhibited alternative dominant clonotypes such as WGFCWPR/ASSRDIPAGAYN and RALKLRKKV/TCSEGQRGQTQY (Figure 6H). Together, these results demonstrate that protective immunity is linked to a focused, clonally expanded CD8^+^ T cells, predominantly residing within defined protection‐associated transcriptional states, rather than a broadly activated but functionally ineffective T cell response.

### 2.7 CD8^+^ T Cell Activation Preferentially Involves Macrophages/Monocytes Crosstalk

**FIGURE 7:**
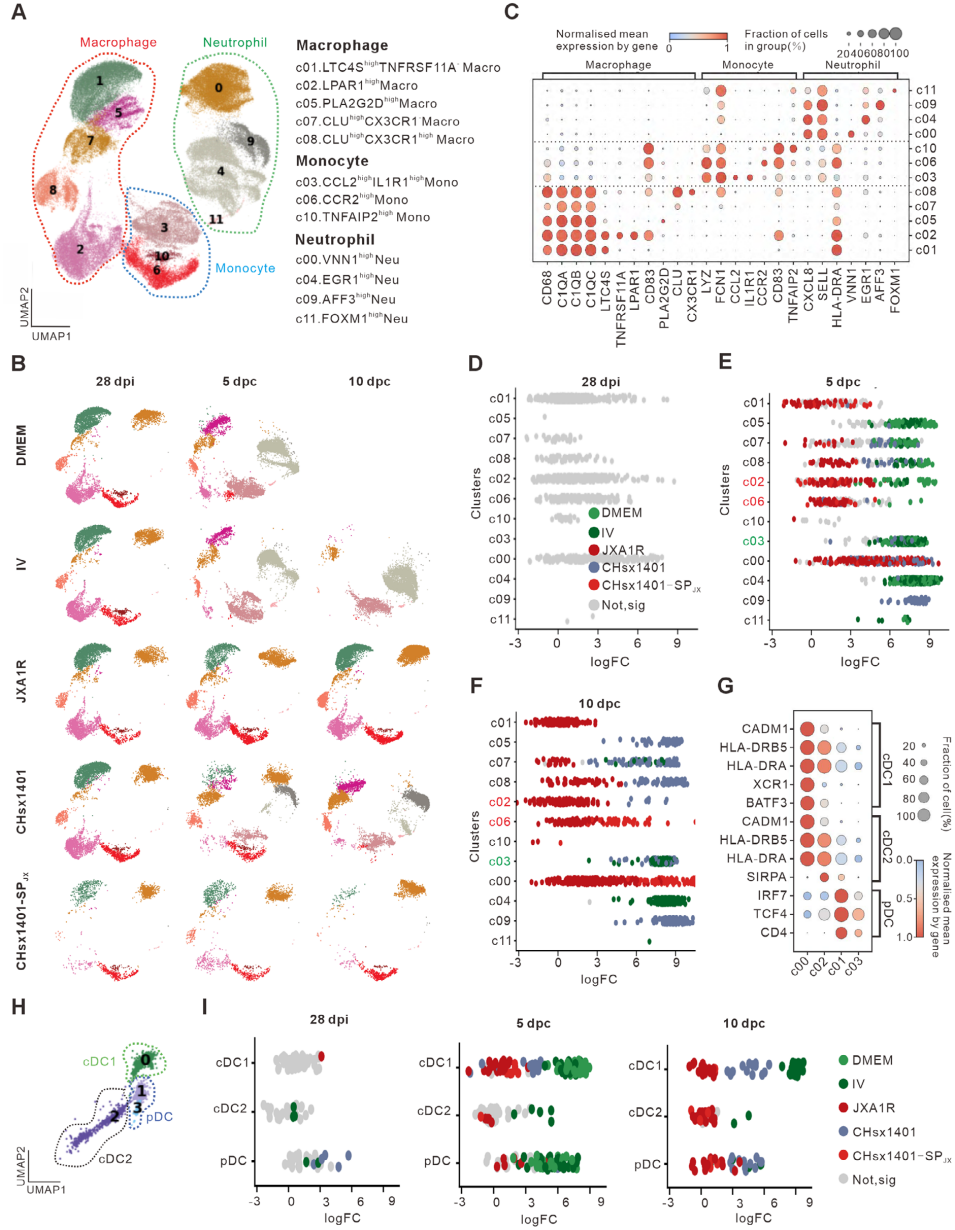
Compositional changes of myeloid cells following challenge. (A) UMAP plot of macrophage/monotyce/neutrophil population with the integrated single cell transcriptomes of 86 039 cells from spleen in all groups. (B) Temporal dynamic changes of macrophage/monotyce/neutrophil populations at indicated timing. (C) Definition of the different macrophage/monotyce/neutrophil cell types. Specific markers used to delineate the individual cell type are indicated and their expression levels represent group‐wise mean log‐normalized values scaled from 0 to 1 for each gene. (D–F) Abundance analysis of significantly enriched macrophage/monocytes/ neutrophil subpopulations in different experimental groups at indicated time points via miloR. Each dot represents a MiloR neighborhood. The logFC reflects the abundance change of subclusters within each B cell clusters in each group that was normalized against that of DMEM at 28 dpi. (G–I) The same as above, except for analysis of DC cells with a total of 8443 cells, including specific markers (G), UMAP (H), and abundance analysis (I) of DC cells.

Given that effective CD8^+^ T cell activation depends on the co‐stimulatory signals from antigen‐presenting cells (APC), we next examined the compositional changes of macrophages, monocytes, neutrophils, DC, and B cells across time points (at 28 dpi, 5 dpc, and 10 dpc). Following challenge, protected animals (JXA1R and CHsx1401‐SP_JX_) showed increased abundance of macrophage (c02, c01, c08) and monocyte (c06) subsets, along with expansion of neutrophils (c00), cDC2, and multiple B cell subsets (c01, c00, c04, and c06) (Figure 7; Figure S8). In contrast, the non‐protected groups (IV and DMEM) showed evidence of macrophage depletion or dysfunction, accompanied by increases in alternative monocyte (c03), cDC1, neutrophil (c04), and B cell subsets (c02, c03, and c05) (Figure 7; Figure S8). The partially protected CHsx1401 group again displayed an intermediate phenotype (Figure 7; Figure S8).

**FIGURE 8:**
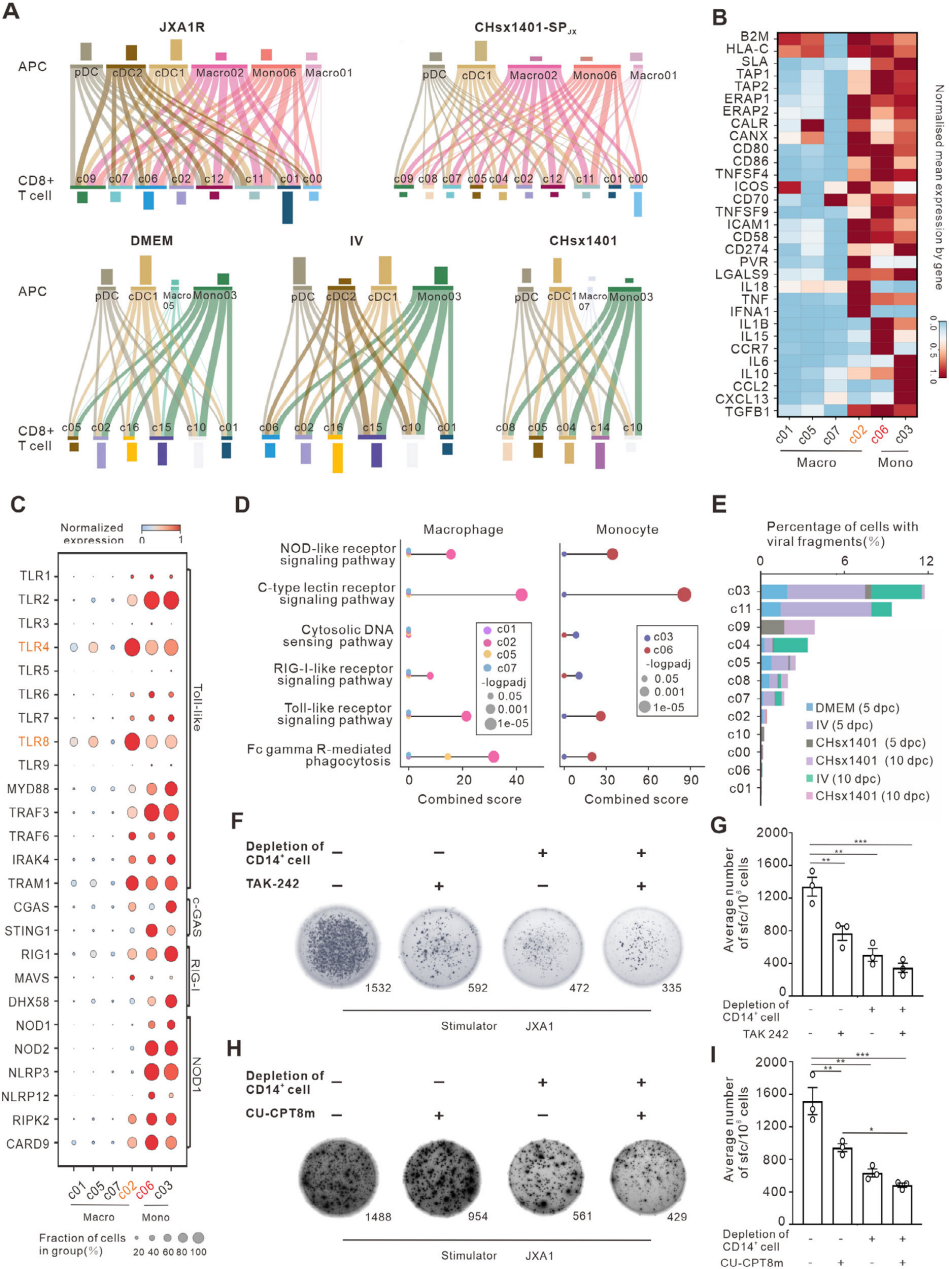
CD8^+^ T cells preferentially receive signals from macrophages /monocytes for activation via TLR4 signaling. (A) Communications between APC and CD8^+^ T cells within each experimental group as inferred by CellPhoneDB. (B) Analysis of the expression level of antigen presentation‐related genes in indicated cell subclusters. (C) Dot plot showing normalized mean expression level of genes involved in innate immunity in indicated subclusters of macrophages and monocytes. (D) The KEGG enrichment analysis of innate immunity‐associated pathways for differentially upregulated genes in the subclusters of macrophages and monocytes. The enrichment level is determined by the number of included genes and their expression levels. (E) Percentage of cells containing virus RNA fragments in the indicated cell clusters. (F, G) Role of TLR4 signaling in T cell activation. The spleen cell mixture from three pigs of JXA1R groups at 5dpc were prepared. The ability to secret IFN‐γ was determined using ELISpot in the presence of TAK‐242, a TLR4 inhibitor or absence of macrophage/monocytes that were depleted by mouse monoclonal antibodies to CD14. The representative ELISpot wells (F) and statistical analysis (G) were presented. (H, I) Role of TLR8 signaling in T cell activation with TLR8 inhibitor. The representative ELISpot wells (H) and statistical analysis (I) were presented. Statistical analysis was performed by two‐tailed Student's t‐test, and error bars indicate means ± standard error of mean (SEM). Asterisks (*) indicate the statistical significance: *, *p* < 0.05; **, *p* < 0.01; ***, *p* < 0.001.

To further investigate functional interactions, we performed ligand–receptor analysis to infer cell‐cell communication networks. While CD8^+^ T cells in all groups received signals from dendritic cells (DC), protected groups (JXA1R and CHsx1401‐SP_JX_) showed unexpectedly enhanced strong interactions with macrophage subset c02 and monocyte subset c06, whereas unprotected/partially protected groups relied more prominently on monocyte c03 (Figure 8A). Notably, interactions between CD8^+^ T cells and neutrophils or B cells (Figure 8A) were minimal across all conditions.

Functional characterization of these APC subsets revealed distinct immunological profiles. In particular, macrophage c02 and monocyte c06, enriched in protected animals, expressed high levels of antigen presentation genes and produced pro‐inflammatory and activating cytokine (e.g., *IL‐18*, *TNF‐α*, *IFNA1*, *IL‐1β*, *IL‐15*). In contrast, monocyte c03, predominant in unprotected groups, was associated with expression of more inhibitory mediators (e.g., *IL‐10*, *CCL2*, *CXCL13*) (Figure 8B; Figure S9A,B).

Collectively, the above findings suggest that protective immunity is associated with a distinct APC microenvironment, characterized by macrophage c02 and monocyte c06 subsets, which provide supportive signals for the activation and functional differentiation of protection‐associated CD8^+^ T cell subsets.

### 2.8 TLR4/8 Signaling in Macrophages/Monocytes Contributes to Optimal CD8^+^ T Cell Activation

We next examined the activation status of major innate immune signaling and interferon‐stimulated gene (ISG) programs across macrophage/monocyte subsets. Although macrophage c02 and monocyte c06 exhibited lower ISG expression compared to monocyte c03, they displayed stronger activation of Toll‐like receptor (TLR) signaling pathways, together with upregulation of genes associated with antigen presentation and adaptive immune priming (e.g., *CD74, GBP1, GBP2, and GBP5*) (Figure 8C,D; Fig. S9A).

Notably, macrophage c02/monocyte c06 contained minimal detectable viral RNA, while monocyte c03 harbored substantially high viral loads (Figure 8E), suggesting that these subsets may represent functionally distinct APC states associated with either effective immune priming or viral permissiveness. Among TLR family members, *TLR4 and TLR8* were prominently expressed in macrophage c02/monocyte c06 (Figure 8C), implicating these pathways in shaping the activation state of these APC populations.

Subsequent loss‐of‐function assays supported this notion. Depletion of CD14^+^ monocytes/macrophages with antibodies to CD14 markedly reduced IFN‐γ secretion by splenocyte cultures upon PRRSV stimulation (Figure 8F,G; Figure S9C), indicating a critical contribution of these cells to T cell activation. Pharmacological inhibition of TLR4 signaling using TAK‐242, a selective TLR4 antagonist, resulted in a significant reduction (> two‐thirds) of IFN‐γ production. Importantly, TAK‐242 had minimal additional effect following CD14^+^ cell depletion (Figure 8F,G), supporting that its effect is primarily mediated through macrophages/monocytes. Similar conclusions could be obtained using the TLR8 inhibitor (CU‐CPT8m) (Figure 8H,I), further indicating that multiple TLR pathways contribute to this process.

We also investigated the specificity of the inhibitors. We found that TAK‐242 selectively inhibited LPS‐induced TNF‐α production (TLR4‐dependent), while CU‐CPT8m selectively inhibited R848‐induced TNF‐α production (TLR8‐dependent). Importantly, neither inhibitor suppressed Poly(I:C) induced IFN‐β expression, indicating that RLR/TLR3‐associated antiviral signaling pathways were not broadly affected (Figure S9D). These results support that the observed effects are pathway‐specific under our experimental conditions.

Together, these findings support a model in which TLR4‐ and TLR8‐dependent signaling in macrophages/monocytes contributes to the optimal activation of CD8^+^ T cells. Rather than acting in isolation, these innate sensing pathways likely function in a coordinated manner to establish an APC state that promotes effective antiviral T cell responses.

## 3 Discussion

**FIGURE 9:**
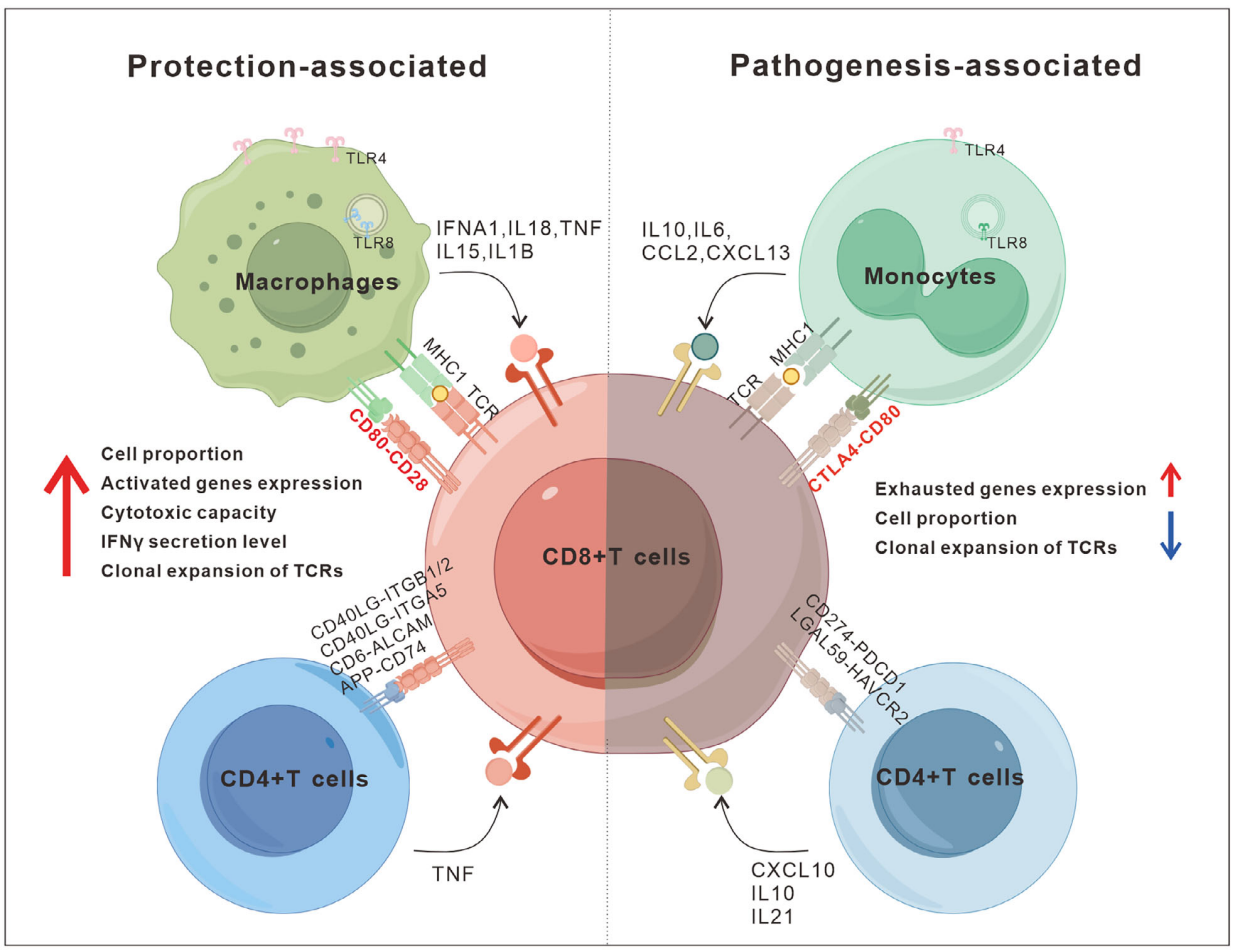
Schematic model summarizing immune features associated with protection and pathogenesis. In the protected group, antigen presentation is associated with specialized subset of macrophages and monocytes characterized by high *TLR4* and pro‐inflammatory signals expression. With appropriate CD4^+^ T cell help, these CD8^+^ T cells acquire robust cytotoxic effector functions and mediate efficient antiviral immunity. In contrast, in unprotected animals, antigen presentation is associated with monocyte populations expressing suppressive and inhibitory signals. CD8^+^ T cells in this context exhibit increased expression of exhaustion‐associated markers, especially *CTLA4*, together with reduced effector functionality. Aberrant CD4^+^ T cell–CD8^+^ T cell communication further reinforces T cell exhaustion, ultimately resulting in impaired antiviral responses and disease progression.

The ever‐increasing genetic diversity and limited cross‐protection efficacy to heterologous strains highlight a central need to define the mechanisms that underpin vaccine‐mediated protection and to understand how viral variations reshape these responses [25, 41]. In this report, by integrating an immunization–challenge model with single‐cell profiling and functional validation, we delineated key cellular features of protective immunity. Specifically, we identify discrete CD8^+^ T cell subsets as correlates of protection, demonstrate their strong association with viral structural proteins (SP)‐dependent responses, and uncover a macrophage/monocyte‐driven activation axis involving TLR4/8 signaling (Figure 9). The relative significance and implications of these findings are discussed below.

A key long‐standing question in PRRSV immunology concerns the relative contribution of T cell subsets to viral clearance. Prior studies have described PRRSV‐responsive T cell populations, including γδ T cells, CD4^+^ T cells, CD4^+^CD8^+^ T cells, and CD8^+^ T cells. However, these conclusions have often relied on PBMC‐based restimulation assays, which lack tissue context and have limited resolution in distinguishing protective versus dysfunctional states [29, 31, 34, 42]. Specifically, γδ T cells exhibit non‐selective cytotoxicity and respond usually around 42–56 dpi [29, 43], a timing rather late when the viremia has already been cleared. In contrast, the responding CD4^+^ T cells are detectable around 14 dpi [29], whereas the responding CTL could be detected around 28 dpi following MLV immunization [26, 29, 44]. By using the immunization‐challenge model, several studies propose that CD4^+^ T cells play an important role as evidenced by their proliferation in response to restimulation by heterologous strains [45, 46], while in another study CD8^+^ T cell responses were found to correlate with the immune protection by using HP‐PRRSV MLV as vaccine and NADC30‐like strain as the challenge virus [32]. Overall, the conflicting conclusions regarding CD4^+^ versus CD8^+^ dominance in protection likely reflect these methodological limitations.

In this study, analyses of splenic immune responses provide multiple lines of evidence supporting the dominant role of CD8^+^ T cells in protective immunity. (i) CD8^+^T cells were the only population that consistently expanded early after challenge in protected animals, coinciding with viremia control (Figures 1E, 2D, 3B), whereas this was not observed in non‐protected groups (Figures 2C,D and 3B). Although the CD4^+^ T cells were noticeably enriched in the protected groups, they were mainly naïve and memory subsets rather than effector populations (Figure 4B,D). (ii) CD8^+^ T cells exhibited stronger cytotoxicity and higher IFN‐γ production upon re‐stimulation as compared to CD4^+^ T cells (Figures 3D,E, 4F, and 5C,E), and depletion of CD8^+^ T cells markedly reduced IFN‐γ secretion in splenocyte cultures (Figure 5E,F; Figure S6). (iii) Specific CD8^+^ T cell effector subsets (c01/c07/c11/c12) were selectively enriched in protected animals and displayed coordinated activation and cytotoxic transcriptional programs (e.g. *CD28*, *PRF1*, *GZMM*, *GZMH*, *GZMK*, etc.) consistent with functional activity. (iv) scTCR‐seq analysis showed that these subsets correspond to the dominant clonally expanded populations in protected animals following challenge (Figure 6F), whereas CD4^+^ T cells, by contrast, remained largely low in clonality, consistent with a helper‐dominant rather than effector‐dominant role (Figure 6F). These findings also explain well previous observations that CD4^+^ T cells can proliferate to different degrees in response to various PRRSV strains [46], in which the clonal proliferation is most likely low clonal and non‐directional (high TCR diversity) according to the finding in this study. (v) Finally, in the gain‐of‐function control, SP exchange in the chimeric virus (CHsx1401‐SP_JX_) reshaped the CD8^+^ T cell landscape to a profile resembling that observed in fully protected animals, in parallel with restoration of clinical protection. Thus, while direct in vivo functional validation (e.g., adoptive transfer, tetramer staining, etc.) is currently limited, these convergent lines of kinetic, functional, transcriptional, clonal, and genetic evidence strongly support that these CD8^+^ T cell populations represent antigen‐experienced, PRRSV‐responsive subsets closely associated with protective immunity.

It should be noted that, although our study defined protection‐associated CD8^+^ T cell states in the spleen, this tissue may not fully represent local immune responses in the lung, which is the primary site of PRRSV replication in porcine alveolar macrophages. In addition, lymphoid tissues, including draining lymph nodes, can also serve as sites of viral replication and immune activation, harboring immune responses that may differ from those observed in the spleen. Therefore, the immune signatures identified here should be interpreted primarily in the context of systemic immunity. Future studies incorporating lung tissue, bronchoalveolar lavage cells, and lymph nodes will be necessary to determine whether the protection‐associated immune states identified in the spleen are similarly represented at local sites of infection and to better understand how systemic and tissue‐resident immune responses cooperate to mediate protection against PRRSV.

Our results also clarify that the presence of cytotoxic gene expression alone is not a sufficient marker of protection or immune activation. In non‐protected groups, CD8^+^ T cells expressed high levels of cytotoxic molecules such as granzymes, yet these cells were characterized by exhaustion‐associated programs dominated by CTLA4 and other inhibitory pathways (Figures 3D and 4F). This suggests that the failure of immune protection is not due to a lack of cytotoxic potential, but rather reflects functional impairment imposed by inhibitory regulatory states. This observation is consistent with findings in chronic infection and tumor immunology, where exhaustion programs can limit functional cytotoxicity despite the preserved transcriptional signatures [47, 48]. A recent report shows that approximately 74% of proteins tend to form aggregates in exhausted T cells, including the key effector granzyme B [49]. The non‐canonical proteotoxic stress response (Proteotoxic Stress Response, Tex‐PSR), triggered by the accumulation of misfolded proteins, is a critical mechanism driving T cell exhaustion. In the case of PRRSV, the enrichment of CTLA4^high^ exhausted CD8^+^ T cell subsets in non‐protected animals, as well as their presence in partially protected conditions, suggests that early induction of inhibitory pathways may restrict the development of effective antiviral immunity, and could serve as an early biomarker for insufficient protection against PRRSV.

A second important observation is that CD8^+^ T cell responses associated with protection are strongly influenced by viral genetic composition, particularly the structural protein‐coding region. Using the SP‐chimeric virus, we observed that replacement of the SP region reshaped the CD8^+^ T cell landscape from a mixed cytotoxic/exhausted profile associated with partial protection toward a phenotype resembling that induced MLV, accompanied by restoration of clinical protection. These findings support a major contribution of SP‐derived antigens to protective T cell responses, consistent with the protection patterns observed in the previous animal experiments [50, 51]. Certainly, our data do not exclude contributions from other viral regions. Notably, even SP exchange restored complete clinical protection, IFN‐γ responses did not fully reach the MLV level upon restimulation (Figure 5A), suggesting that additional epitopes likely contribute the magnitude and breadth of recall responses. It should be pointed out that IFN‐γ production represents only one aspect of T cell activation. The observation that IFN‐γ responses in the CHsx1401‐SP_JX_ group did not fully reach the levels seen in the JXA1R group suggests that protection is not solely determined by the magnitude of ex vivo cytokine responses. Instead, multiple factors, including the quality, timing, and differentiation states of CD8^+^ T cell responses, as well as clonal expansion and early viral control, may collectively enable CHsx1401‐SP_JX_ to reach a protective threshold. For future studies, a direct comparison of SP‐ versus nsp‐driven immune effect would further strengthen this mechanistic interpretation, such as cell stimulation with defined nsp‐ and SP‐derived peptide pools. In addition, construction of reciprocal chimeric virus will allow a more complete dissection of the relative contributions of structural and non‐structural regions to protective immunity.

In addition to cellular immunity, we also examined B cell activation states using the scRNA‐seq dataset (Figure S8). B cell populations in both diseased and protected groups showed evidence of activation and differentiation, including dynamic changes in genes associated with B cell responses. Notably, B cell populations c00 (memory cells) and c01 (naïve B cells) exhibited a significant loss following challenge in non‐protected groups (DMEM and IV groups) (Figure S8B), whereas these populations were relatively preserved in protected animals. In contrast, the subset c03 showed a significant increase in the same group in the diseased animals. Although the functional significance of these B cell subsets remains unclear, these findings suggest that PRRSV infection differentially reshapes B cell states depending on protection outcome. Neutralizing antibody titers remained relatively low and delayed in this model (<1:10; Figure 1G), consistent with previous reports that PRRSV neutralizing antibodies often arise late and at modest titers [26, 52], arguing against a dominant role in early protection at the analyzed time points. In contrast, protection in our model showed a much stronger association with early expansion, clonal activation, and functional differentiation of CD8^+^ T cell subsets. Nevertheless, because structural proteins are major targets of antibody responses and PRRSV non‐structural proteins, particularly nsp2, are known to induce early but predominantly non‐neutralizing antibodies [52, 53], our findings do not exclude a supportive role for humoral immunity, particularly in longer‐term, strain‐specific, or synergistic protective responses.

Another notable finding is the role of macrophage/monocyte subsets in supporting CD8^+^ T cell activation. While dendritic cells are classically considered as the primary APC for CD8^+^ T cell priming [54], our analysis revealed strong interactions between CD8^+^ T cells and specific macrophage/monocyte subsets (macrophage c02 and monocyte c06) (Figure 8A) in protected animals. These subsets expressed high levels of genes related to antigen presentation machinery and immune activation cytokines (Figure 8B) and, strikingly, contained minimal viral RNAs (Figure 8E), suggesting a functionally competent APC state. In contrast, macrophage (c05)/monocyte subset (c03) enriched in non‐protected animals carried high viral loads and expressed inhibitory signals such as *IL‐10* and chemokines associated with cellular immune suppression (Figure 8B,E), indicating a less favorable environment for effective T cell activation. Functional experiments further showed that depletion of CD14^+^ cells or inhibition of TLR4/8 signaling significantly reduced IFN‐γ production, supporting a role for these pathways in facilitating CD8^+^ T cell activation (Figure 8F–I). Together, these findings suggest that a TLR4/8‐dependent myeloid niche provides a permissive activation context that enables protective, non‐exhausted CD8^+^ differentiation, whereas alternative myeloid states promote inhibitory signaling and exhaustion.

In summary, our work provides in vivo, single‐cell‐resolved insights into the immune responses associated with PRRSV vaccine‐induced protection. Our findings support a model in which effective immunity involves coordinated interactions between viral antigen composition, innate immune activation, and CD8^+^ T cell differentiation. These results have implications for vaccine design, including optimization of antigen selection and the potential use of adjuvants that enhance beneficial innate signaling while limiting inhibitory pathways. Future studies incorporating additional tissues and higher‐resolution antigen‐specific approaches, such as tetramer‐based assays, and improved porcine antibody panels for protein‐level validation of defined immune subsets, will further refine the mechanisms underlying protective immunity against PRRSV.

## 4 Experimental Section

### 4.1 Ethics Statements

The animal experiment was carried out according to the Chinese Regulations of Laboratory Animals—The Guidelines for the Care of Laboratory Animals (Ministry of Science and Technology, People's Republic of China) and Laboratory Animal‐Requirements of Environment and Housing Facilities (GB 14925‐2010, National Laboratory Animal Standardization Technical Committee). The study protocol (license number: AW82115202‐2‐03) was approved by the Laboratory Animal Ethical Committee of China Agricultural University.

### 4.2 Cell Culture and Antibodies

Primary porcine alveolar macrophages (PAMs) derived from 1‐month‐old SPF pigs were cultured in RPMI 1640 (Gibco, #61870044) containing 10% fetal bovine serum (FBS; Gibco, #16140071) and penicillin (50 U/mL) and streptomycin (50 mg/mL) at 37°C with 5% CO_2_, whereas MARC‐145 cells (ATCC, CRL‐12231) were maintained in Dulbecco's modified Eagle's medium (DMEM; Gibco, #12491015). Mouse antibodies to porcine pe‐cy7‐labeled CD3ε (BD, #561477), FITC‐labeled CD3ε (Thermo Fisher, #ZK4541661), PE‐labeled CD8α(BD, #559584), PE‐labeled CD8β(BD, #561484), APC‐labeled CD4α (Thermo Fisher, #MA5‐28730), PE‐Cy7‐labeled CD4α (BD, #561473), FITC‐labeled CD8α (BD, #551303) and PE‐labeled CD14 (Abcam, #AB186689) were purchased from indicated companies.

### 4.3 Vaccine and Viruses

Highly pathogenic porcine reproductive and respiratory syndrome live attenuated vaccine (Strain JXA1R) purchased from Yangzhou Weike Co. The inactivated form of JXA1R were prepared with 1‰ formaldehyde for 24 h and adjuvated with ISA 61VG (Seppic) in a water in oil form. The Chinese highly pathogenic PRRSV strain JXA1 (Gen Bank accession no: EF112445) and the NADC30‐like strain CHsx1401 (Gen Bank accession no: KP861625) and the chimeric virus CHsx1401‐SP_JX_ used in this study have been described previously [15, 55, 56].

### 4.4 Animal Experiments

The 27‐day‐old SPF British Large White piglets. Piglets were confirmed negative for PRRSV, pseudorabies virus (PCV2), porcine circovirus type 2 (PRV), classic swine fever virus (CSFV), and African swine fever virus (ASFV) by commercial ELISA kits and RT‐PCR. The animals were housed in different rooms of the animal facility and allowed to adapt to the environment for 3 days prior to experiments. For the immunization/challenge experiment, pigs were randomly divided into six different groups: JXA1R MLV (*n* = 16), CHsx1401‐SP_JX_ (*n* = 16), JXA1R IV (*n* = 16), CHsx1401 (*n* = 16), DMEM (*n* = 16), and Blank group (*n* = 3). The piglets were immunized via intramuscular route with the respective viruses at a dose of 2 × 10^5^ TCID_50_, while the DMEM group were inoculated with DMEM and there was no any treatment for the Blank group. At 28 dpi, all piglets, except for the blank group, were challenged with JXA1 at a dose of 2 × 10^6^ TCID_50_ and euthanized at 10 dpc. The animals were monitored daily for clinical signs and rectal temperatures. The detailed scoring system was performed according to the method described previously [57]. The blood was sampled at indicated time points to investigate viremia, total antibody, and neutralizing antibody responses. Meanwhile, three pigs per group were euthanized and spleen was collected at indicated time points (28 dpi, 5 dpc, and 10 dpc) for single‐cell RNA sequencing, except for the DMEM group, for which samples were only collected at 28 dpi and 5 dpc. Pigs that were euthanized for scRNA‐seq at 28 dpi and 5 dpc were excluded from the mortality analysis. Mortality was monitored in groups of 10 pigs each. The lungs and lymph nodes were collected for pathological evaluation and tissue viral load quantification. The standard scoring system of lung gross pathology has been described elsewhere [57]. Specifically, the gross pathology was evaluated from 0 to 100 according to the lesion area of each lung lobe. The accessory lobes were assigned 5 points each, and 27.5 points (15 for dorsal and 12.5 for ventral) for each of the right and left caudal lobes to reach a total of 100 points. The scores of lung microscopic lesions were blindly evaluated from 0 to 4 according to the severity of the interstitial pneumonia (0 = no microscopic lesions; 1 = mild; 2 = moderate multifocal interstitial pneumonia; 3 = moderate diffusive interstitial pneumonia; 4 = severe interstitial pneumonia).

### 4.5 Quantitative PCR (qPCR)

The viral RNA levels of tissues were quantitated by qPCR as described previously [56]. Total RNAs were extracted with TRIzol (Thermo Fisher, #15596026) according to the manufacturer's instructions. cDNAs were synthesized by reverse transcription using the FastKing reverse transcription kit (Tiangen, #KR116). To measure the viral tissue load, a pair of specific primers and a specific probe were used to amplify JXA1 nsp9, and a standard curve was generated for the nsp9 gene by plotting log10 copy number against the cycling threshold (CT) value. Quantitative PCR (qPCR) was performed with Super Real PreMix containing the probe (Tiangen, #FP206) according to the manufacturer's recommendations. PCR was performed in a 20‐µL reaction containing 200 ng of cDNA, 0.3 µM gene‐specific primer, 0.2 µM probe, and 10 µL of Super Real PreMix. PCR parameters were 95°C for 15 min and 40 cycles of 95°C for 10 s and 60°C for 32 s. As for verification of the specificity of TAK‐242 and CU‐CPT8m. Cells were treated with LPS (1000 ng/mL), Poly(I:C) (1000 ng/mL) alone or in combination with TAK‐242 (5 µM) or CU‐CPT8m (5 µM) for 12 h. The relative mRNA levels of TNF‐α and IFNβ were normalized to GAPDH and compared with those in the agonist‐only stimulation group. Primer sequences used are listed in Table S1.

### 4.6 Collection of Spleen Samples and Isolation of Splenic Cells

After euthanasia, splenic samples were cut into approximately 1 mm^3^ pieces in RPMI‐1640 medium and enzymatically digested with 3 mg/mL Collagenase D (Roche, #11088882001). The tissues were then incubated at 37°C for 30 min with intermittent agitation followed by centrifugation at 400 × g for 5 min to collect the cell pellet, and resuspended in RPMI 1640 medium with 10% FBS. The cells were filtered through 70 µm strainers and subject to red blood cell lysis before enrichment. The cell viability for each sample was found to exceed 90% as examined by AO/PI staining. The cells were utilized for either immediate assays (e.g., scRNA‐seq, cytotoxicity, etc.) or subject to cryopreservation. For scRNA‐seq, the scRNA‐seq libraries were generated using Chromium Single Cell 3′ Library & Gel Bead Kit v.2 (10× Genomics); for TCR analysis, the libraries were generated by Chromium Next GEM Single Cell 5′ Kit v2 (10 × Genomics) and pig‐specific V(D)J primers (Genergy, #SP‐2400‐0004), according to the manufacturer's protocol. Libraries were sequenced on Illumina HiSeq X Ten. For flow cytometry analysis, the cell pellets were washed twice with PBS and then re‐suspended in sorting buffer (PBS with 2% FBS) for further staining.

### 4.7 Sorting of Specific Immune Cells

Single‐cell suspensions were generated from spleen. CD3^+^ T, CD8α^+^ T, CD8β^+^ T, CD4^+^ T, CD8α^+^ T‐depleted, CD4^+^ T‐depleted, CD14^+^ myeloid cells, and CD14^+^‐depleted cells were sorted by BD Aria III cell sorter after staining with corresponding mouse antibodies to pig CD3, CD4, CD8α, CD8β, or CD14. The staining was performed following the manufacturer's instruction (BD Biosciences). Specifically, the cells were washed with flow cytometry staining buffer (3% FBS in PBS) for 5 min at 1500 rpm, and were incubated in PBS containing 10% pig negative serum on ice for 15 min to block nonspecific binding. Surface staining was performed at 4°C for 30 min with the fluorescently labeled antibodies. CD3^+^, CD4^+^, CD8α^+^, and CD8β^+^ T cells were stained with antibodies to porcine CD3, CD4α, CD8α, and CD8β, respectively. The gating strategy was sequentially defined as total cells, single cells, live cells, CD3^+^ cells, which were further subdivided into CD4^+^, CD8α^+^ or CD8β^+^ T cell populations. Monocytes were stained with antibodies to porcine CD14. Sorted cells were cultured in RPIM1640 medium (10% FBS, 1% penicillin‐streptomycin, 25 mM HEPES, 1% non‐essential amino acid, and 0.1% β‐mercaptoethanol). At least 100,000 cells were collected by BD Aria III cell sorter and analyzed using FlowJo software (BD Biosciences).

### 4.8 Cytotoxicity Analysis of T Cells

T cells, CD4^+^ T cells, and CD8^+^ T cells were isolated using antibodies to CD3, CD4, or CD8α/β by FACS. 1 × 10^5^ T cells were cultured in RPIM 1640 medium and activated with 10 U/mL porcine IL‐2 (Beyotime, #P6461) for 24 h. 2 × 10^3^ autologous monocytes from PBMCs were isolated using CD14 antibody and inoculated with PRRSV strain JXA1 at a multiplicity of infection (MOI) of 1 for 12 h. The isolated T cells were plated in round‐bottomed 96‐well plates with PRRSV‐infected monocytes at the CTL‐to‐target ratios 50:1. The co‐cultures were incubated at 37°C for 8 h, and the percentage of target cell lysis was determined using the LDH cytotoxicity assay kit (Beyotime, #C0016) following the manufacturer's instructions.

### 4.9 Serum Neutralization Assay

The method has been described previously. Briefly, serum samples were heat inactivated at 56°C for 30 min and then diluted at twofold series with DMEM and incubated with an equal volume (50 µL) of PRRSV strain JXA1 at an amount of 100 TCID_50_ at 37°C for 1 h. Afterward, the mixture was incubated with MARC‐145 cells cultured in a 96‐well plate for another 1 h at 37°C. Then the cells were washed three times with PBS, followed by the addition of fresh DMEM media supplemented with 2% FBS. After 24 h, PRRSV‐positive cells were detected by IFA with antibodies to PRRSV N protein. The titer of neutralizing antibodies (NA) was calculated using the Reed‐Muench method [58].

### 4.10 ELISpot Assay

The level of IFN‐γ was determined using the porcine IFN‐γ ELISpot kit (Mabtech, #3130‐4APW‐10) following the manufacturer's instructions. 2 × 10^6^ cells/well were used to reduce sampling error and improve the reproducibility of low‐frequency events. This condition was selected to ensure sufficient sensitivity for detecting recall responses. Briefly, 96‐well plates were pre‐coated overnight with anti‐porcine IFN‐γ monoclonal antibody. Splenic single‐cell suspensions were then plated at 2 × 10^6^ cells per well in RPMI 1640 medium. For virus preparation, each PRRSV strain was propagated on a large scale, and culture supernatants were collected. Cell debris was removed by centrifugation at 5000 × g for 20 min. The clarified supernatants were then precipitated with 8% PEG 8000 and 0.2 M NaCl at 4°C, followed by centrifugation at 12 000 × g for 30 min. The viral pellet was resuspended in PBS and further concentrated and purified by sucrose‐gradient ultracentrifugation. The visible viral band between the 30% and 40% sucrose layers was collected, diluted, and subjected to an additional round of ultracentrifugation to remove residual sucrose. The final purified virions were resuspended in PBS. For re‐stimulation, purified PRRSV virions were added at an MOI of 2. The final virus inoculum volume was standardized to 10 µL per well; when the calculated virus volume was less than 10 µL, PBS was added to bring the volume to 10 µL. Because the purified virions were finally resuspended and diluted in PBS, control wells received an equivalent volume of PBS as the matched vehicle control. Cells were re‐stimulated with the indicated PRRSV virions for 24 h at 37°C. IFN‐γ‐secreting cells were detected using a biotinylated anti‐pig IFN‐γ detection antibody and visualized using an ImmunoSpot image analyzer. For the TLR‐4 or TLR‐8 loss‐function assay, the CD14‐deleted cells were pre‐incubated with TAK‐242 (MCE, #243984‐11‐4) or CU‐CPT8m (MCE, #HY‐112050) at a final concentration of 5 µM for 2 h, followed by the addition of virus, and ELISpot was then performed.

### 4.11 ScRNA‐Seq Data Processing

Three pigs from each group were harvested at the indicated time points (28 dpi, 5 dpc, and 10 dpc) for single‐cell RNA sequencing. Notably, samples from the DMEM group were only collected at 28 dpi and 5 dpc. Except for the SP group, in which spleen single‐cell suspensions from three pigs were pooled for sequencing, all other groups were sequenced individually for each pig. The scRNA‐seq data were aligned to the sus scrofa 11.1 genome assembly and gene annotation (Ensembl release 112) using Cell Ranger (v8.0.1), with the JXA1 viral genome. Preliminary filtered feature‐barcode matrices produced by Cell Ranger were used for quality control on a per‐sample basis. Specifically, low‐quality genes and cells were filtered out by removing predicted doublets (Scrublet with data‐driven thresholds), retaining genes expressed in more than three cells and cells with 500–25 000 UMIs, >250 detected genes, and <20% mitochondrial transcripts. Subsequent analysis was performed using the OmicVerse (v1.6.10) and Scanpy (v1.10.3) frameworks [59, 60]. After log‐normalization, highly variable genes were identified using analytic pearson residuals selecting 3000 highly variable genes [61]. Principal component analysis (PCA) was performed, and the top 50 principal components were retained for downstream analyses and visualization with Uniform Manifold Approximation and Projection (UMAP). For clustering, a neighborhood graph was constructed using sc.pp. neighbors, and cells were clustered into subgroups using the Leiden algorithm [62]. Multiple resolution parameters (0.1, 0.2, 0.5, 1.0, and 2.0) were tested, and a resolution of 0.5 was selected based on its ability to clearly separate major cell types, as defined by canonical marker genes. B cells were marked by *CD79B* [63]; NK cells by *KLRB1*, *NCR1*, and PRF1 [64]; T cells by *CD2*, *CD3G*, *CD4*, *CD8A*, and *TRDC* [65, 66]; macrophages by *CD68*, *C1QA*, *C1QB*, and C1QC [67]. Monocytes expressed *LYZ* and FCN1 [68]. Neutrophils showed high *CXCL8* and *SELL* but lacked *SLA‐*DRA [69]. Dendritic cells expressed *FLT3*, with *IRF7* marking pDCs and *CADM1* marking cDCs [70]. To enable marker‐based functional annotation and downstream analysis, porcine Ensembl gene IDs were converted to human gene symbols using orthology information from the PANTHER knowledgebase (v19.0) [71].

### 4.12 Subset‐Specific Reclustering

To resolve intra‐lineage heterogeneity, CD8^+^ T cells, CD4^+^ T cells, B cells, and myeloid populations (including monocytes, macrophages, and neutrophils) were extracted and re‐analyzed individually, following the same overall pipeline as described above. For each subset, 2,000 highly variable genes were selected, and the top 30 components were retained. Subcluster annotation was based on the expression of differentially expressed genes with high specificity and functional relevance, including those related to cytokine signaling, cytotoxic activity, proliferation, and lineage‐defining transcriptional programs. CD8^+^ T cell subclusters were defined using marker genes such as *LEF1*, *NAV3*, *BCL2*, *TCF7*, *IL7R*, *ZNF683*,  *TNFSF9*,  *NCR1*, *KLRD1*, *PRF1*, *GZMA*, *GZMB*, *GZMK*,  *GNLY*, *PDCD1*, *CTLA4*, and *TOP2A* [72, 73, 74]. CD4^+^ T cell subsets were annotated based on the expression of  *LEF1*, *CCR7*, *SELL*, *NAV3*, *IL7R*, *CD44*, *CD9*, *TBX21*, *IL12RB2*, *IL10*, *GATA3*, *BCL6*,  *CXCR5*, *RORC*, *FOXP3* and *IL2RA* [65, 75, 76]. Myeloid subpopulations were distinguished using markers including * ITGA4*, *EGR1*, *AFF3*, *FOXM1*, *CCR2*, * C5AR1*, *CCL2*, * IL1R1*, *TNFAIP2*,  *LTC4S*, *TNFRSF11A*, *LPAR1*, *CX3CR1*, *CLU*, and *HAVCR2* [77, 78]. B cell subsets were characterized by the expression of *CXCR4*,  *RNF150*,  *TNFRSF1B*, * IL10*,  *CXCL10*,  *ITGAD*,  *FCER1G*,  *IL6R*,  *S100A10*,  *BST1*,  *GCSAM*,  *TOP2A*, and *IL16* [79, 80]. Clusters without clear lineage identity or showing mixed patterns were labeled as unknown and excluded from downstream analysis.

### 4.13 Differential Expression and Functional Enrichment Analyses

Differentially expressed genes (DEGs) between cluster pairs, as well as between selected clusters and the remaining cells, were identified using the pyDEG module from OmicVerse, with significance assessed using the Wilcoxon rank‐sum test. Significant DEGs were defined as those with a false discovery rate (FDR) < 0.05. For functional enrichment, KEGG pathway analyses were conducted using the enrich function in gseapy, with FDR < 0.05 [81]. Gene sets were obtained from the Molecular Signatures Database.

### 4.14 Differential Cell Abundance Analysis of Immune Cells

Differential abundance of various types of immune cells was analyzed using milopy (v0.1.1) [82]. A KNN graph was constructed using the top 2,000 highly variable genes and the first 50 principal components with the sc.pp.neighbors function from Scanpy. Representative neighborhoods were assigned using make_nhoods, and the cell counts per sample within each neighborhood were obtained with count_nhoods. Differential abundance testing was then performed using DA_nhoods, using a generalized linear model (GLM) with the design formula 1 + group. All comparisons were performed using DMEM group samples at 28 dpi as the baseline. Neighborhoods with a FDR < 0.1 were considered significantly differentially abundant and colored according to the group in which enrichment occurred.

### 4.15 Single Cell V(D)J Sequencing Data Analysis

TCR reads were assembled into contigs using the Cell Ranger V(D)J de novo assembly. Due to the incomplete annotation of porcine V(D)J germline sequences, contig annotation was performed with reference to the method described by Gu et al. [83]. Specifically, TCR β chain contigs were aligned to the pig TRB reference from IMGT using IMGT/V‐QUEST, and CDR3 regions were obtained from the V‐QUEST output. For TCR α chains, contigs were mapped using MMseqs2 to the pig TRAJ reference from IMGT and TRAV sequences available in GenBank [84], and CDR3 regions were identified based on conserved alignment boundaries. Only cells with both α and β chain contigs were retained. Contig chain annotation information was integrated into a Python dictionary format and added to the observation field of each cell in the AnnData object. Clone size was defined as the number of cells sharing the same CDR3 α and β pair. Shannon entropy was used to measure the diversity of TCR clonotypes within each cluster, normalized for the number of clonotypes. Gini index was calculated to quantify the distribution of clone sizes, reflecting the degree of clonal expansion within the cluster. Clusters from the 5′ mRNA and V(D)J datasets were mapped to those from the 3′ dataset based on shared differentially expressed and marker genes.

### 4.16 Cell‐Cell Communication Analysis

Ligand‐receptor interactions between clusters was inferred using the CellPhoneDB (v5.0.1) [85]. The ligand‐receptor reference database was curated by integrating CellPhoneDB and CellChat (v2.1.2) to increase interaction coverage [86]. As CellPhoneDB estimates communication strength using mean gene expression, small clusters with a few highly expressing cells may yield inflated scores. To reduce this bias, interactions were evaluated only in clusters in which the fraction of ligand‐ or receptor‐expressing cells exceeded a defined threshold. Thresholds were set to 0.05 for CD8^+^ T cell subsets and 0.1 for CD4^+^ T cells and myeloid populations.

### 4.17 Statistical Analysis

GraphPad Prism version 7.0 (La Jolla, CA, USA) was used for the statistical analyses by a two‐tailed unpaired Student's t‐test. The asterisks indicate the statistical significance: NS, no significance; *, *p* < 0.05; **, *p* < 0.01 ***, *p* < 0.001. Error bars indicate means ± standard error of mean (SEM).

## Author Contributions

**Chen Wang**: validation, visualization. **Bolun Zhou**: validation, visualization. **Siang Chen**: validation, formal analysis, data curation, visualization. **Zhenhua Xie**: validation, visualization. **Meng Wang**: validation, visualization. **Jie Li**: supervision, funding acquisition, resources, conceptualization. **Peng Gao**: project administration, supervision. **Jianjun Luo**: supervision. **Hanchun Yang**: supervision, funding acquisition, resources, conceptualization. **Dongdong Zhang**: supervision, funding acquisition, conceptualization. **Maolin Li**: validation, visualization. **Runsheng Chen**: supervision, funding acquisition. **Jun Han**: conceptualization, supervision, funding acquisition, project administration, methodology, resources, writing – review and editing, investigation. **Hailin Zhang**: validation, visualization. **Can Kong**: conceptualization, methodology, investigation, validation, formal analysis, data curation, funding acquisition, visualization, project administration, writing – original draft.

## Conflicts of Interest

The authors declare no conflicts of interest.

## Supporting information




**Supporting File**: advs76732‐sup‐0001‐SuppMat.pdf.

## Data Availability

The raw 10X Genomics scRNA‐seq data of this study are openly available in National Genomics Data center at https://ngdc.cncb.ac.cn/gsa/, reference number CRA034558.
