# Supplementary material for: Integrated Single‐Cell and TCR Profiling Reveals Protection‐Associated CD8+ T Cell Subsets Linked to Viral Control in PRRSV
Source: Adv Sci (Weinh). 2026 Jul 29:e76732. Online ahead of print. doi: 10.1002/advs.76732 (PMC13418511; doi:10.1002/advs.76732)
Supplement: Supplementary file 1 — Supporting File: advs76732‐sup‐0001‐SuppMat.pdf. [file ADVS-9999-e76732-s001.pdf]

Supporting Information

Integrated Single-Cell and TCR Profiling Reveals Protection-Associated CD8$^+$ T Cell Subsets Linked to Viral Control in PRRSV

Can Kong[#], Siang Chen[#], Maolin Li, Meng Wang, Hailin Zhang, Bolun Zhou, Zhenhua Xie, Chen Wang, Peng Gao, Jianjun Luo, Hanchun Yang, Runsheng Chen, Dongdong Zhang*, Jie Li*, Jun Han**

[#] These authors contribute equally to the paper

*Corresponding authors

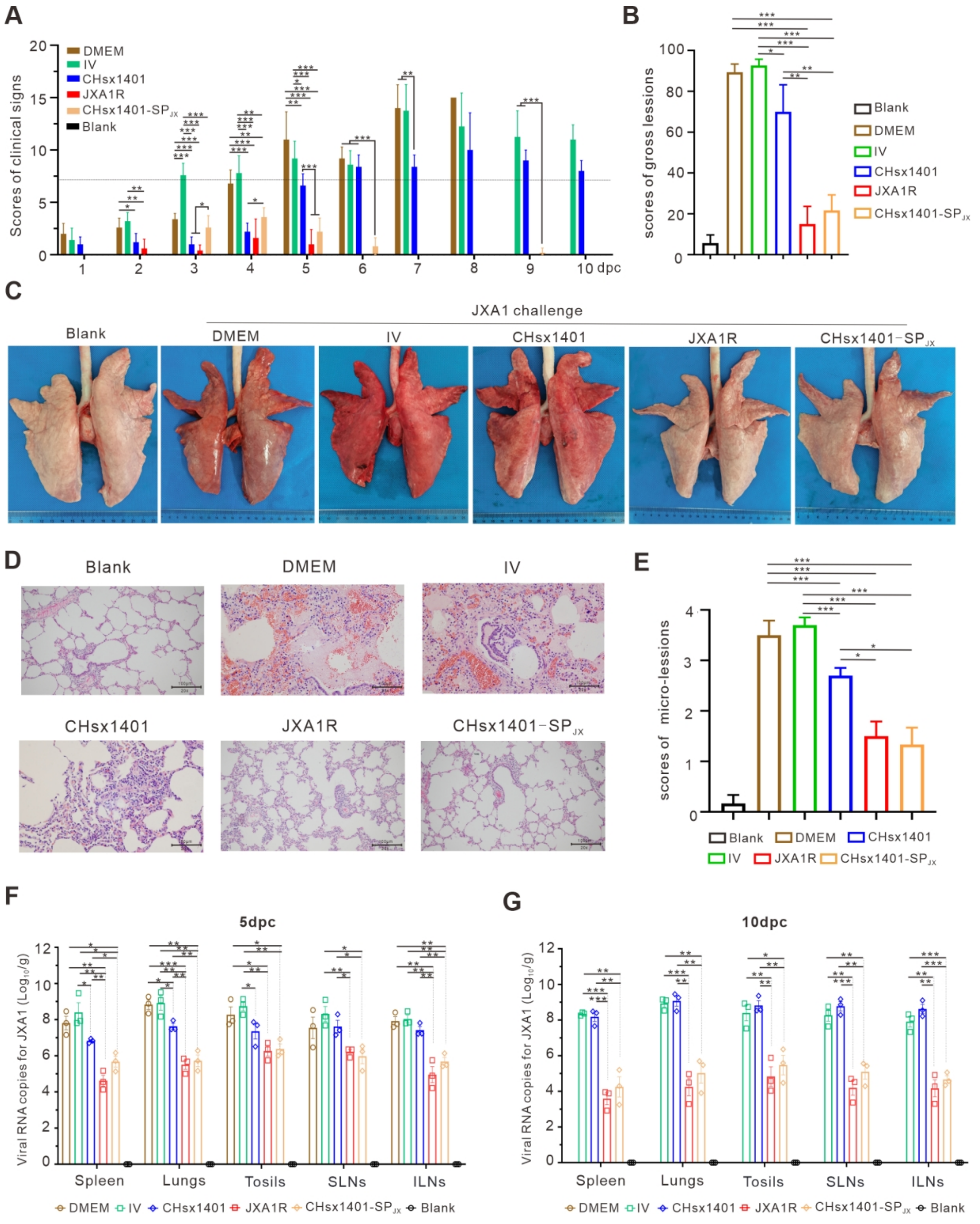

Figure S1. Pig clinical manifestations and viral tissue load following challenge by JXA1. A) Clinical mental scores of piglets. The clinical scoring included the gross clinical score (GCS), respiratory clinical score (RCS), and nervous signs score (NSS). Total scores for each piglet represented the sum of GCS, RCS, and NSS. **B)** Statistical analysis of lung lesions of each group at 10 dpc. **C)** Representative gross lung lesions of each group at 10 dpc. D) Representative images of microscopic lung lesions (H&E staining) at 10 dpc. E) Mean scores of microscopic lung lesions of each group. **F,G)** Viral load in lungs and secondary lymphoid tissues at different days post challenge by absolute qPCR using primers targeting nsp9-coding region. Statistical analysis was performed by two-tailed Student' s t-test and error bars indicate means ± standard error of mean (SEM). Asterisks (*) indicate the statistical significance: *, P < 0.05; **, P < 0.01; ***, P < 0.001.

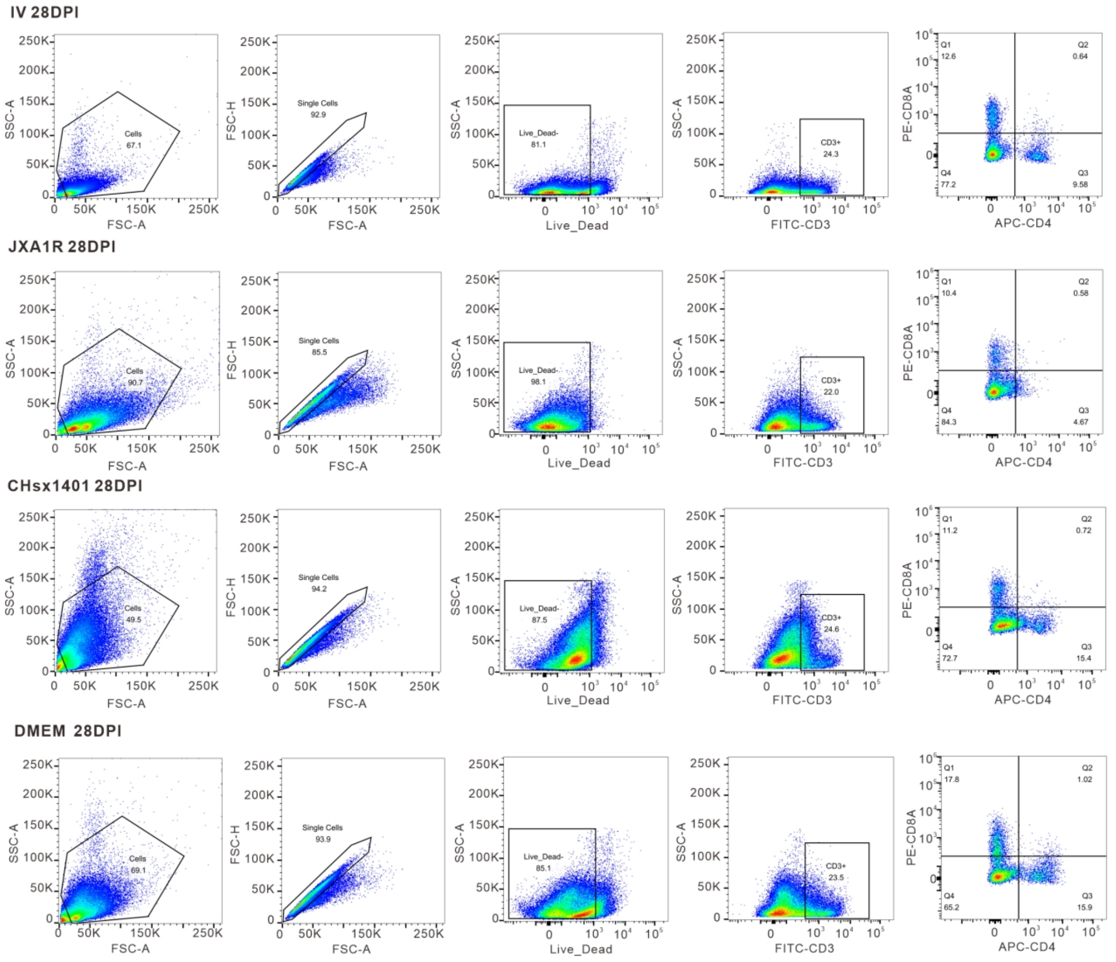

Figure S2. Flow cytometric analysis of the changes in CD4⁺ and CD8⁺ T cells in each group at 28 DPI. Splenic single-cell suspensions were blocked and stained with flow cytometry antibodies against FITC-CD3E, APC-CD4, and PE-CD8α, followed by flow cytometric analysis for the proportion changes of cell subsets. Total lymphocytes were identified based on forward scatter area (FSC-A) and side scatter area (SSC-A), excluding debris events. Next, singlet cells were gated using FSC-A versus FSC-H to remove doublets and cell aggregates. Viable cells were then selected by excluding Live/Dead-positive events. From the live singlet population, $CD3^+$ T cells were gated based on FITC-CD3E staining. Finally, the $CD3^+$ T cell population was further analyzed for CD4 and CD8α expression using APC-CD4 and PE-CD8, allowing separation into $CD8α^+CD4^−$, $CD4^+CD8α^+$, $CD4^+CD8α^−$, and $CD4^−CD8α^−$ T cell subsets.

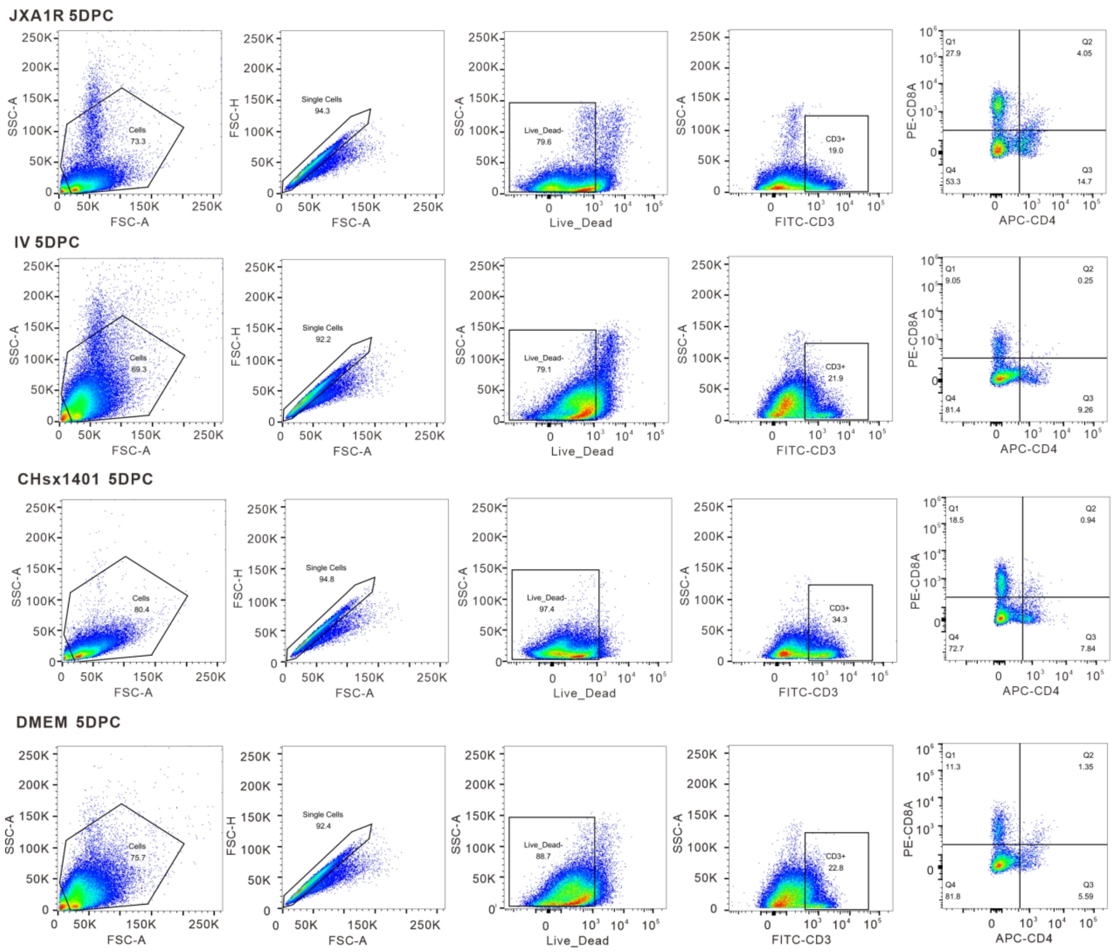

Figure S3. Flow cytometric analysis of the changes in CD4$^+$ and CD8$^+$ T cells in each group at 5 DPC. The gating strategy was sequentially defined as total cells, single cells, live cells, CD3$^+$ cells, which were further subdivided into CD4$^+$ and CD8α$^+$ T cell populations.

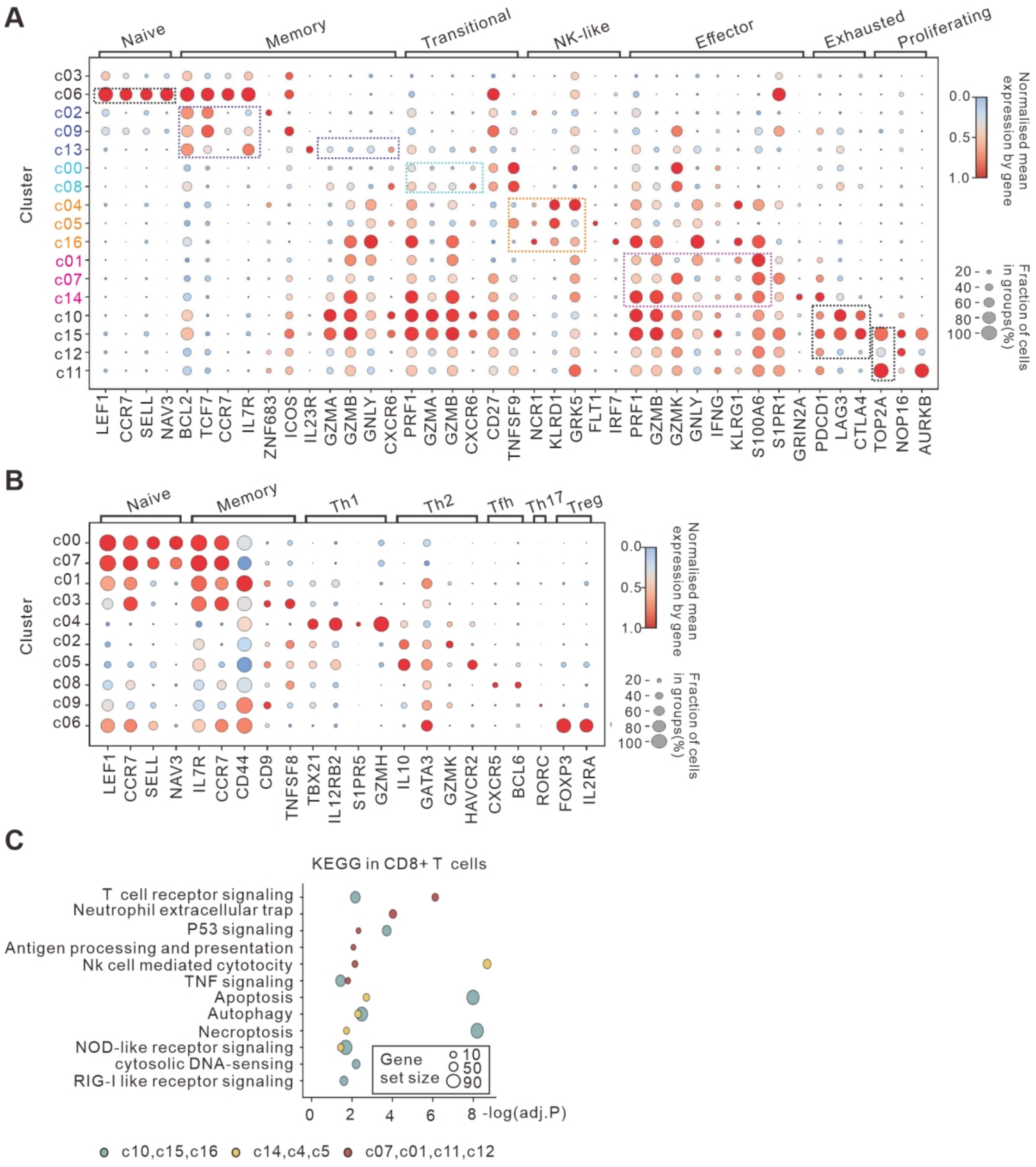

Figure S4. Definition of T cell subsets. A,B) Dot plot showing normalized mean expression levels of marker genes for CD8⁺ (A) and CD4⁺ (B) T cell subclusters. **C)** The KEGG enrichment analysis of immune-related pathways for differentially upregulated genes among CD8⁺ T cell populations.

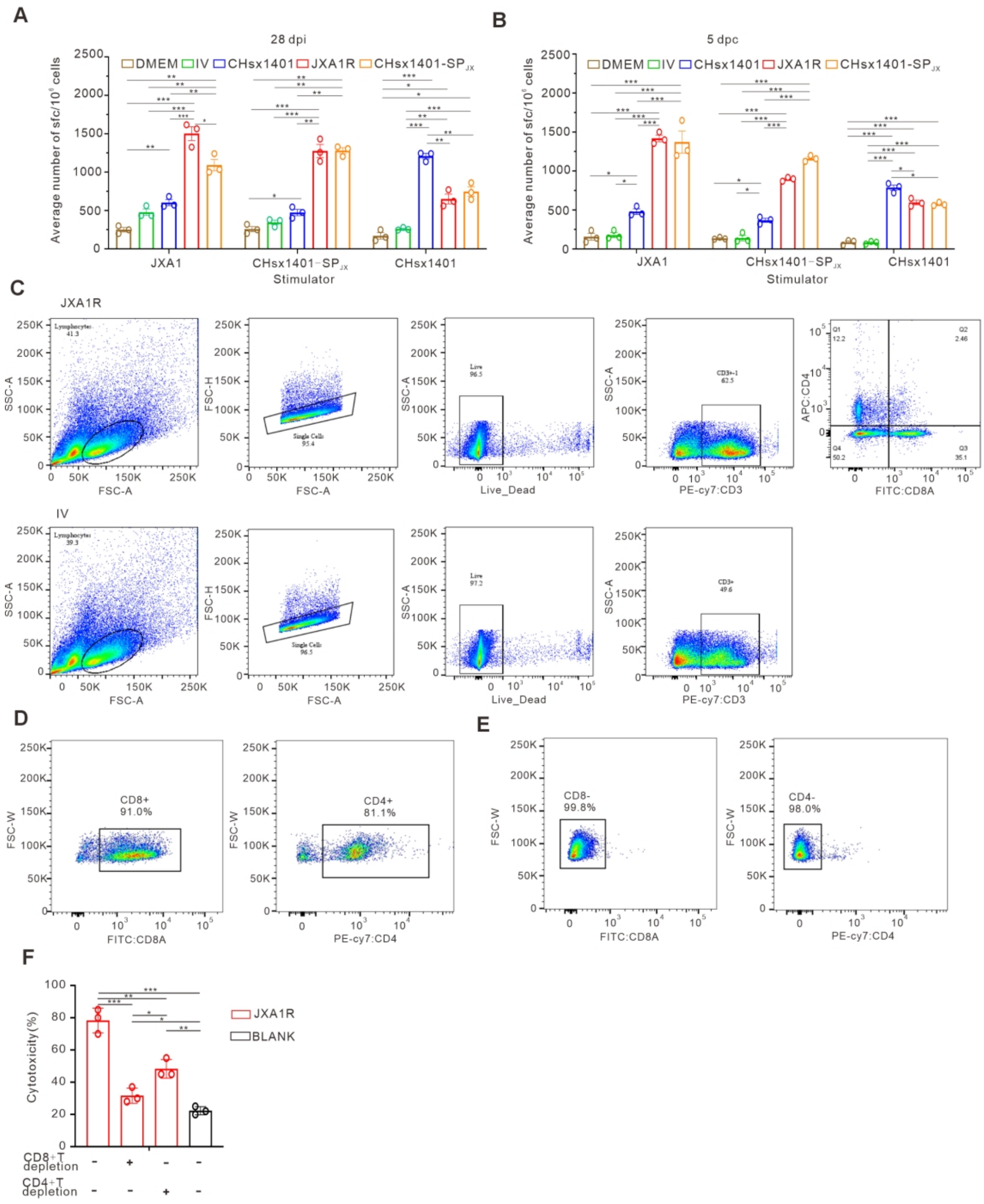

Figure S5. Evaluation of T cell status before and post challenge. A,B) Quantitative analysis of IFN-γ secretion in the ELISpot assay of T cells from different groups at at 28 dpi (A) and 5 dpc (B) restimulated with indicated viruses. **C)** FACS sorting of T cell populations from indicated experimental groups used for *in vitro* cytotoxicity analyses. The gating strategy was sequentially defined as total cells, single cells, live cells, $CD3^+$ cells, which were further subdivided into $CD4^+$ and $CD8\alpha^+$ T cell populations. **D)** Purifity analysis of sorted $CD8\alpha^+$ and $CD4^+$ T cell populations for *in vitro* cytotoxicity analyses. **E)** Purifity analysis of $CD8\alpha^+$-delepted and $CD4^+$-delepted T cell populations for ELISpot. **F)** The loss-of-function assay to evaluate the contributions of $CD4^+$ and $CD8\alpha^+$ T cells by cytotoxicity assay. Statistical analysis was performed by two-tailed student's t-test and error bars indicate means ± standard error of mean (SEM). Asterisks (*) indicate the statistical significance: *, $P < 0.05$; **, $P < 0.01$; ***, $P < 0.001$.

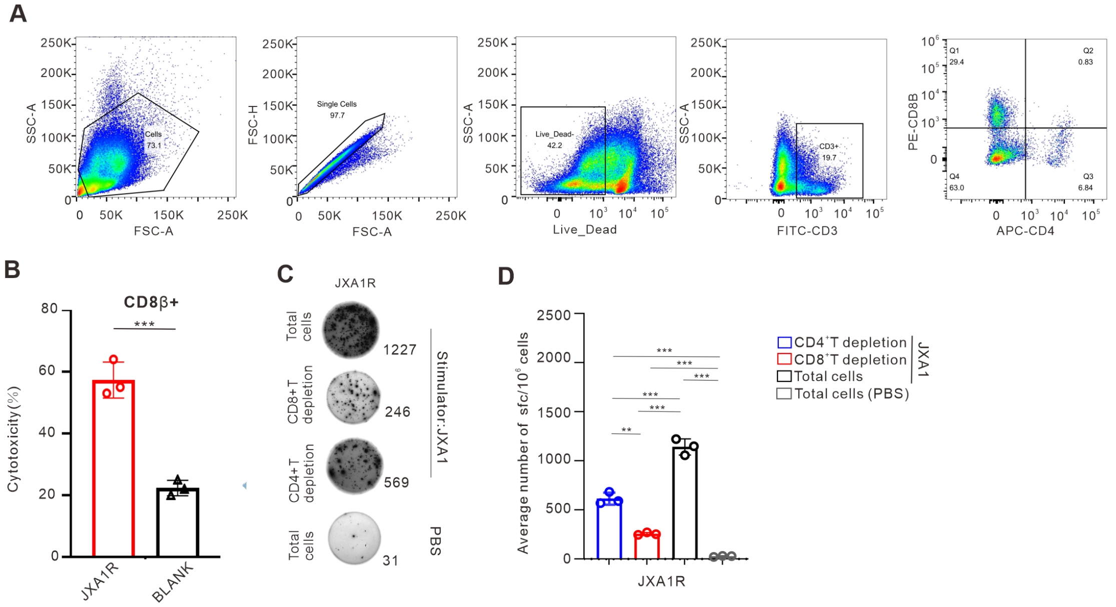

Figure S6. Analysis of the activity of Tcells using CD8β antibody. A) FACS sorting of T cell populations from indicated experimental groups by flow cytometry using anti-CD8β antibodies. The gating strategy was sequentially defined as total cells, single cells, live cells, $CD3^{+}$ cells, which were further subdivided into $CD4^{+}$ and $CD8β^{+}$ T cell populations.. **B**) The cytotoxic activity of CD8β cells from the JXA1R group against PRRSV-infected cells was evaluated. **C,D**) The loss-of-function assay to assess contribution of $CD4^{+}$ and $CD8β^{+}$ T cells by ELISpot assay. (C) Representative pictures of ELISpot. (D) Quantitative analysis. Statistical analysis was performed by two-tailed student's t-test and error bars indicate means ± standard error of mean (SEM). Asterisks (*) indicate the statistical significance: *, P < 0.05; **, P < 0.01; ***, P < 0.001.

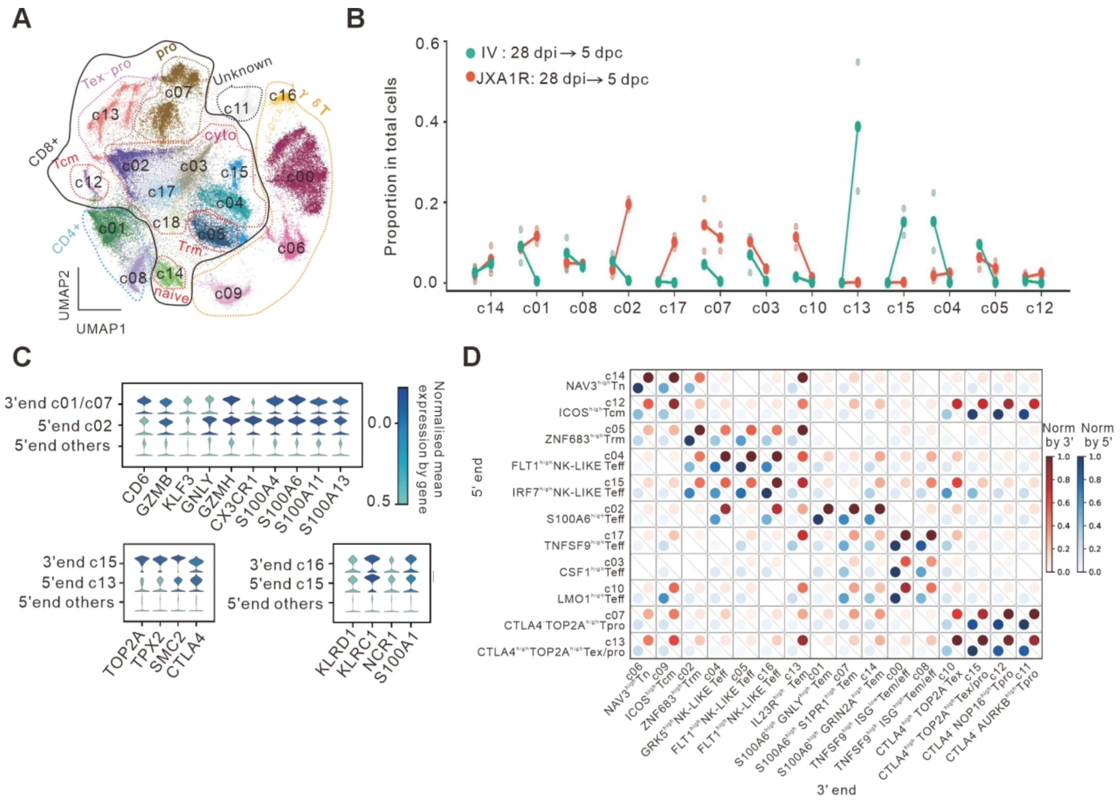

Figure S7. Overview of 5' scRNA sequencing and TCR analysis. A) UMAP projection of all αβ T cells by 5' scRNA seq. Each dot represents a single cell, colored according to cell type. **B)** Proportional changes over time (28 dpi and 5 dpc) of CD4^{+} and CD8^{+} T cell subpopulations of JX1R and IV groups. **C)** Stacked violin plot of median expression of representative differentially expressed genes in 5' transcriptome clusters c02, c13, and c15 versus 3' transcriptome clusters c01, c07, c15, and c16. **D)** Correspondence of all cell subpopulations between 5' scRNA seq and 3' scRNA seq, calculated based on the proportions of shared DEGs relative to each dataset.

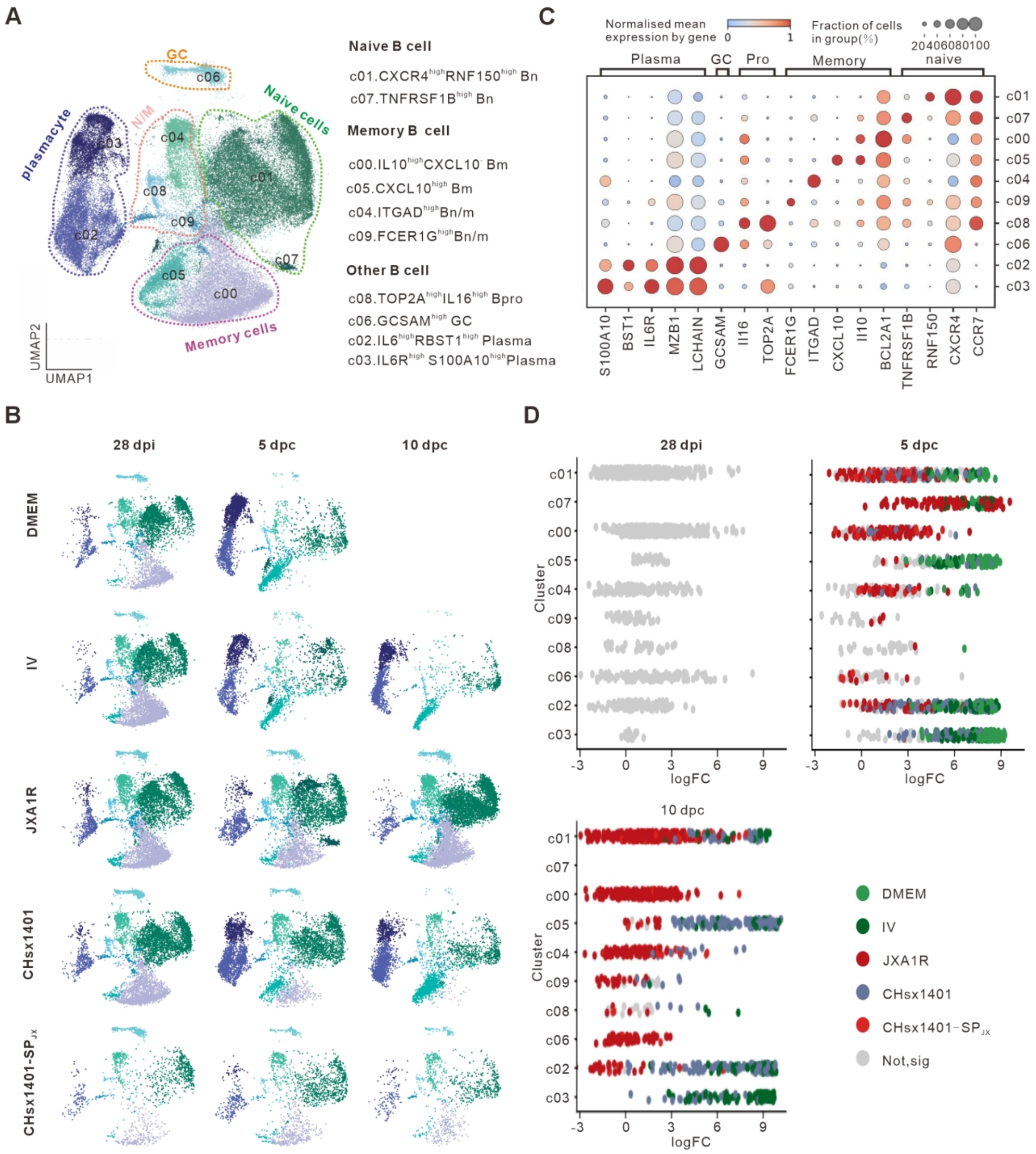

Figure S8. Compositional changes of B cells following viral challenge. A) UMAP plot of B cell subclusters. A total of 10 subclusters marked with different colors were identified and their corresponding cellular markers for definition were indicated. **B)** Temporal dynamic changes of B cell subpopulations by UMAP plot. **C)** Definition of the different B cell types. Specific markers used to delineate the individual cell type are indicated and their expression levels represent group-wise mean log-normalized values scaled from 0 to 1 for each gene. **D)** Abundance analysis of significantly enriched B cell subpopulations in different experimental groups at indicated time points via miloR. Each dot represents a MiloR neighborhood. The logFC reflects the abundance change of subclusters within each B cell clusters in each group that was normalized against that of DMEM at 28 dpi. Colored neighborhoods indicate significant differential abundance (FDR < 0.1).

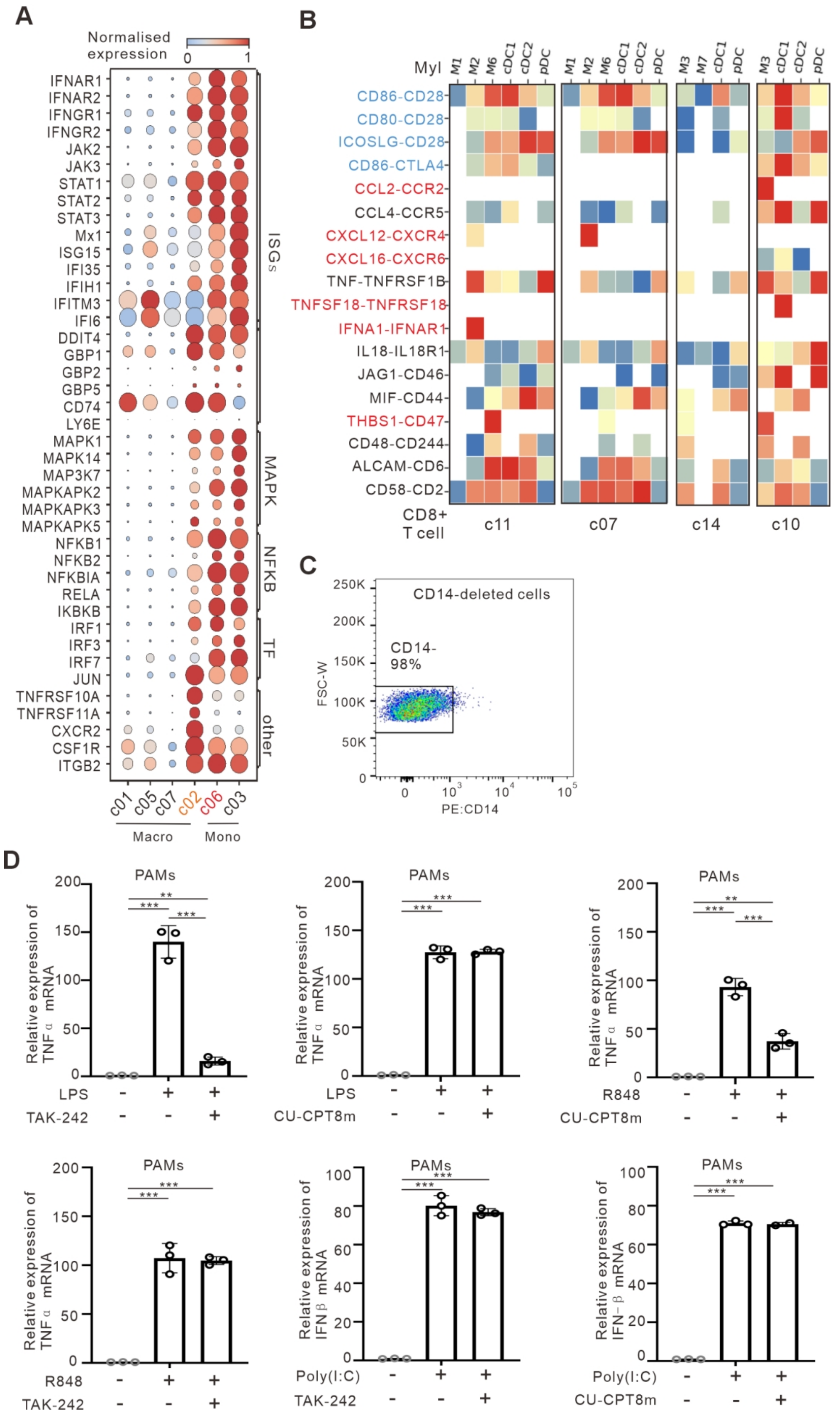

Figure S9. Immunological features of APC subsets. A) Dot plot showing normalized expression level of genes involved in innate immunity and signal transduction in indicated subclusters of macrophages and monocytes. The expression level was calculated using

group-wise mean log-normalized values scaled from 0 to 1 for each gene. **B)** Analysis of most often used interaction pairs for immune activation between indicated APC and $CD8^+$ T cell clusters. The color indicates the interaction strength. **C)** Purity analysis of sorted CD14-deleted cells from splenic cells in JXA1R group at 5 dpc. **D**) Verification of the specificity of TAK-242 and CU-CPT8m in PAMs using quantitative PCR (qPCR). Cells were treated with LPS (1,000 ng/mL), Poly(I:C) (1,000 ng/mL), or R848 (1,000 ng/mL) alone or in combination with TAK-242 (5 μM) or CU-CPT8m (5 μM) for 12 h. The relative mRNA levels of TNF-α and IFNβ were normalized to GAPDH and compared with those in the agonist-only stimulation group. Statistical analysis was performed by two-tailed Student's t-test and error bars indicate means ± standard error of mean (SEM). Asterisks (*) indicate the statistical significance: *, $P < 0.05$; **, $P < 0.01$; ***, $P < 0.001$.

Table S1. Primer sequence for qPCR.

Name	Sequences (5'→3')
JXA1 nsp9-F	CTGCGATCGATCCACACCTG
JXA1 nsp9-R	GCGTGACCAGTAAGTCGTGG
JXA1 nsp9-probe	VIC-CCTGTGCTGAGGAGCATCTACC-MGB
TNF-α-F	CGTTGTAGCCAATGTCAAAGCC
TNF-α-R	TGCCCAGATTCAGCAAAGTCCA
IFN-β(sus)-F	GCATCTCGTGGATAATCAATAC
IFN-β(sus)-R	TACCAACAAAGGAGCAGCAAT
IFN-β (monkey)-F	GCTCTCCTGTTGTGCTTCTCCAC
IFN-β (monkey)-R	CAATAGTCTCATTCCAGCCAGTGC
GAPDH (monkey/sus)-F	TGATGACATCAAGAAGGTGGTGAAG
GAPDH (sus)-R	TCCTTGGAGGCCATGTGGACCAT
GAPDH (monkey)-R	TCCTTGGAGGCCATGTGGGCCAT

F: forward primer; R: reverse primer
